# Oxytocin, Erectile Function and Sexual Behavior: Last Discoveries and Possible Advances

**DOI:** 10.3390/ijms221910376

**Published:** 2021-09-26

**Authors:** Maria Rosaria Melis, Antonio Argiolas

**Affiliations:** Department of Biomedical Sciences, Section of Neurosciences and Clinical Pharmacology, University of Cagliari, 09124 Cagliari, Italy; argiolas@unica.it

**Keywords:** oxytocin, erectile function, sexual behavior

## Abstract

A continuously increasing amount of research shows that oxytocin is involved in numerous central functions. Among the functions in which oxytocin is thought to be involved are those that play a role in social and sexual behaviors, and the involvement of central oxytocin in erectile function and sexual behavior was indeed one of the first to be discovered in laboratory animals in the 1980s. The first part of this review summarizes the results of studies done in laboratory animals that support a facilitatory role of oxytocin in male and female sexual behavior and reveal mechanisms through which this ancient neuropeptide participates in concert with other neurotransmitters and neuropeptides in this complex function, which is fundamental for the species reproduction. The second part summarizes the results of studies done mainly with intranasal oxytocin in men and women with the aim to translate the results found in laboratory animals to humans. Unexpectedly, the results of these studies do not appear to confirm the facilitatory role of oxytocin found in male and female sexual behavior in animals, both in men and women. Possible explanations for the failure of oxytocin to improve sexual behavior in men and women and strategies to attempt to overcome this impasse are considered.

## 1. Introduction

Oxytocin is the neurohypophyseal peptide that is well known for its hormonal role in lactation and parturition and was discovered and synthesized together with the other neurohypophyseal peptide arginine-vasopressin in the 1950s by the Nobel Price Du Vigneaud and coworkers [1,2] (see Table 1 for the amino acid sequence of neurohypophyseal peptides and a few of their peptidic and non-peptidic analogues). Oxytocin is found in the same amount in female and male mammals in the magnocellular and parvocellular neurons, whose cell bodies are in the paraventricular (PVN) and supraoptic (SO) nuclei of the hypothalamus. These neurons project to the neurohypophysis, from which the peptide is released through blood circulation. The PVN and surrounding periventricular structures also contain the cell bodies of parvocellular oxytocinergic neurons that project to extra-hypothalamic brain areas (i.e., medial preoptic area, ventral tegmental area, hippocampus, amygdala, septum, bed nucleus of the stria terminalis, medulla oblongata, and spinal cord) (see [3,4,5,6,7,8,9,10,11,12,13]). Since their discovery in the 1980s (see [14,15]), these central oxytocinergic neurons have been involved in numerous central functions, i.e., memory; learning; affiliative and socio-sexual behaviors, from erectile function to copulatory behavior; yawning, and many others, from the control of pain and feeding behavior to drug dependence (see [3,4,5,6,16,17,18,19,20,21,22,23,24]). In the last fifteen years, there have been many reports that have appeared and that are still appearing that have reported the effects of intranasal oxytocin, a delivery route believed to allow the crossing of the blood–brain barrier by the peptide, which can then reach and act at the level of the central nervous system (see [25]). However, some skepticism for the blood–brain barrier crossing and survival abilities of the peptide in blood circulation is still present among researchers in this field (see [26,27,28,29,30] and Section 8). Nonetheless, these reports further stressed the involvement of oxytocin in the functions recalled above in human beings, i.e., trust, empathy, facial expression recognition, decision making, and so on (see [31,32,33,34,35] and references therein). Although caution is always required when such a large amount of findings appear suddenly (see [28,36,37,38,39]); these studies have allowed the suggestion that oxytocin may have a role in the treatment of mental diseases such as schizophrenia, autism, drug addiction, eating disorders, fibromyalgia, and other neuropsychological disturbances [40,41,42,43,44,45,46,47,48], often with contradictory and negative results [49,50,51,52].

Erectile function and sexual behavior are among the first central functions in which oxytocin was discovered to be involved in the 1980s. In fact, since the first pioneering studies conducted in the 1960s that showed a facilitatory effect of oxytocin on male sexual behavior by revealing that intravenous-systemic oxytocin was either capable of decreasing the latency to the first ejaculation and of retarding the sexual exhaustion of male rabbits paired with a receptive female [53] as well as of ameliorating psychogenic impotence in a limited number of human patients [54], numerous other studies have confirmed that oxytocin is deeply involved in erectile function and copulatory behavior. In line with these results, plasma oxytocin has been found to be increased during sexual activity, mainly at ejaculation [55,56], and by the manipulation of the breast and of the genitalia, which usually take place during sexual intercourse [57]. The sexual effects of oxytocin were only definitively ascertained in the1980s, when picomole amounts of oxytocin given intracerebroventricularly were found to be able to induce penile erection [58,59] and to enhance copulatory behavior in male rats [60] and lordosis in female rats [61,62]. These sexual effects were found to be mediated by uterine-type oxytocinergic receptors [63], see ([64,65,66,67,68] and references therein) (for a review on oxytocin receptors, see [69,70]). Importantly, the pro-erectile effect of oxytocin was ascertained to be testosterone-dependent since it was eliminated by hypophysectomy or castration and was reinstated by supplementation with testosterone or its metabolites, estradiol and 5α-dihydro-testosterone given together [71]. The aim of this work is to review studies on the role of oxytocin in erectile function and sexual behavior, from penile erection and copulation in laboratory animals to sexual intercourse in humans, including the most recent ones in which oxytocin was administered intranasally in humans, in order to provide an updated picture of the most recent discoveries and new possible advances that may be useful for realizing new strategies for the treatment of erectile dysfunction or other related sexual disorders based on the modulation of central oxytocin neurotransmission activity. The studies selected for review were chosen from Pubmed and Google Scholar medlines using search terms such as oxytocin; erectile function; and male and female sexual behavior in several animal species, i.e., rat, mouse, hamster, vole, monkey, non-human primate, quail, zebra finch, and man and woman only on the basis of the presence of experiments aimed at studying the sexual role of oxytocin in the Results section. No method was used to validate and/or summarize the evidence for and against the facilitatory role of oxytocin in erectile function and sexual behavior given by the selected studies.

## 2. Oxytocin and Erectile Function

**Figure 1:**
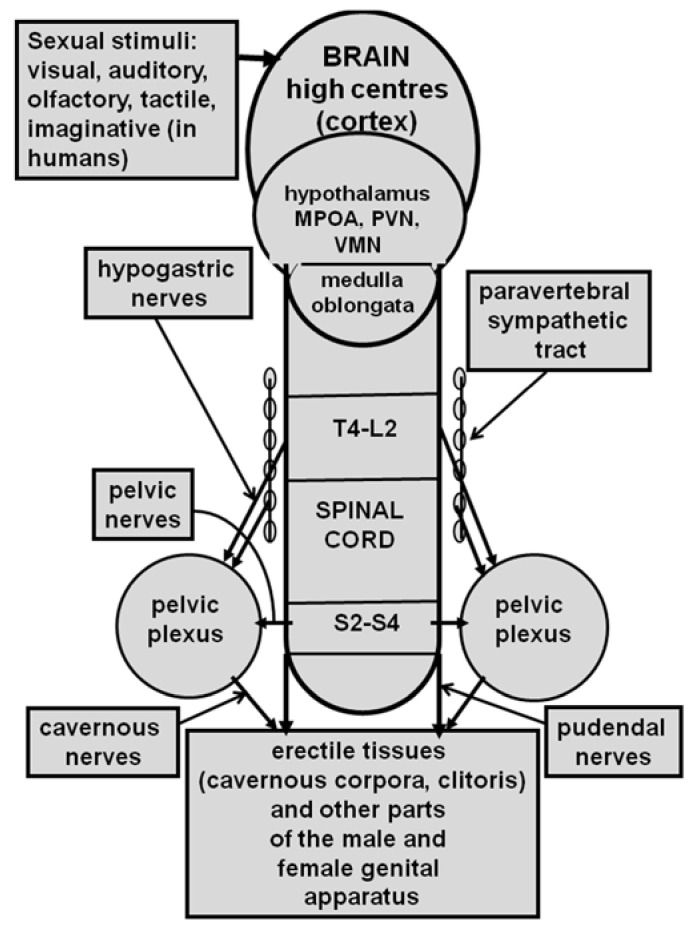
A synthetic view of the neural pathways controlling the genital apparatus in male and female mammals. When visual, auditory, olfactory, and tactile as well as imaginative (in humans) sexual stimuli reach the central nervous system high centres, this activates still unknown neural pathways inducing penile erection in males and clitoris erection and vaginal lubrication in females, enabling sexual intercourse. These pathways run from the brain, especially from the hypothalamus and its nuclei (medial preoptic area—MPOA, paraventricular nucleus—PVN, ventromedial nucleus—VMN), through the medulla oblongata and the spinal cord, and to the genitalia in males and females. The latter are innervated by the pudendal nerves originating from the sacral tract of the spinal cord (S2-S4) that contain the primary afferent sensory and motor pathways to the penis in males and to the clitoris in females and by cavernous nerves that control the primary efferent sympathetic and parasympathetic pathways that arise from the pelvic plexuses. These receive neural inputs by the hypogastric nerves that arise from the thoraco-lumbar T4-L2 spinal tract and from the pelvic nerves arising from the sacral S2-S4 spinal tract. Pelvic plexuses also receive post-gangliar fibres arising from the paravertebral sympathetic ganglia of the thoraco-lumbar T11-L2 spinal tract.

Penile erection results from a complex neural interaction between the central and peripheral nervous system, which causes muscle and vascular changes in the erectile tissues of the male genital apparatus (cavernous corpora, corpus spongiosum, and other perineal muscles, i.e., the “elevator ani” muscle when this is present). This is further complicated by humoral and endocrine influences, especially by testosterone and its metabolites, that are either at central or peripheral levels (see [72,73,74,75]). Penile erection can occur not only during sexual behavior but also in other contexts, i.e., after manipulation of the genitalia; during sleep or erotic fantasies in men or in male rats when put in the presence of an inaccessible receptive female rat (non-contact erections); or after treatment with different drugs (i.e., dopamine agonists, serotonin agonists, nitric oxide (NO) donors, phosphodiesterase inhibitors, soluble guanylate cyclase activators, RhoA-Rho kinase inhibitors, etc.) and neuropeptides (i.e., adrenocorticotropin (ACTH)-melanonocyte stimulating hormone (α-MSH)-related peptides, oxytocin, hexarelin analogues, etc.) acting in the central nervous system or peripherally (see [64,65,67,68,75,76,77,78,79,80,81,82,83,84,85,86]). Depending on the context in which penile erection takes place, it is usually recognized that different neural and/or humoral endocrine mechanisms may participate at the central and peripheral levels in terms of its regulation, often in a very complex manner (see Figure 1 for a simplified view of the central and peripheral neural pathways controlling erectile function and sexual behavior). As recalled above, in the 1980s, oxytocin given centrally (ICV) in picomole amounts was found to be able of inducing penile erection in male rats [58] by acting on uterine-type oxytocinergic receptors [63], (see [64,65,67,68] and references therein) and in a testosterone-dependent manner, an ability that was eliminated by hypophysectomy or castration and that was restored by supplementation with testosterone or its metabolites given together [71].

### 2.1. Oxytocin-Induced Penile Erection: Sites of Action in the Brain

Since the original discovery in 1986 that oxytocin induces penile erection (and yawning) when injected into the PVN and in the dorsal hippocampus (CA1 field) of male rats [87], it is now known that oxytocin also induces penile erection when injected into other areas of the rat brain. Those that have been identified so far are the ventral tegmental area, the ventral subiculum of the hippocampus, the posteromedial nucleus of the amygdala, the bed nucleus of the stria terminalis, the medial preoptic area, and the spinal cord.

#### 2.1.1. PVN

**Figure 2:**
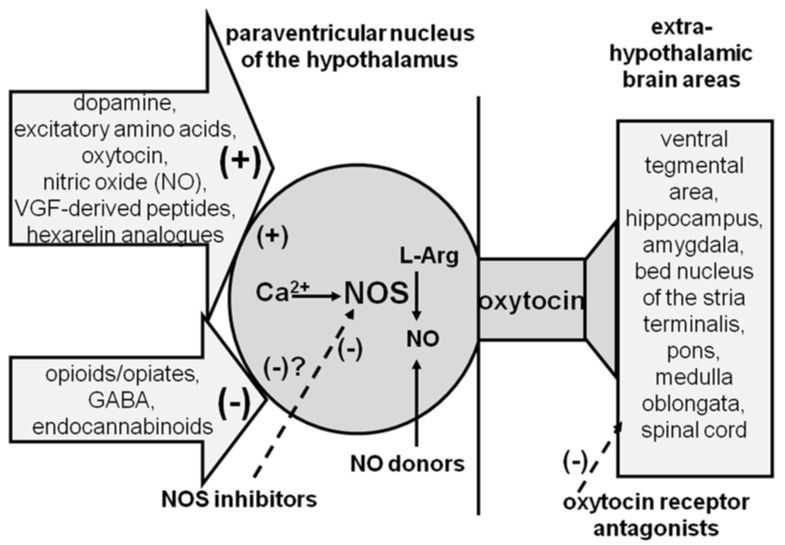
Oxytocin induces penile erection and copulatory activity by activating its own neurons in the rat PVN. Briefly, oxytocin acts on its own receptors in the cell bodies of oxytocinergic neurons; this increases the influx of Ca^2+^ ions in the cell bodies of oxytocinergic neurons and causes the activation of nitric oxide synthase (NOS), a Ca^2+^ calmodulin-dependent enzyme that converts the amino acid L-arginine (L-Arg) to nitric oxide (NO). NO, in turn, activates oxytocinergic neurons, leading to the release of oxytocin in the spinal cord and extra-hypothalamic brain areas. This causes penile erection and facilitates sexual behavior by a mechanism not involving the guanylate cyclase-cyclic guanosine monophosphate (GC-cGMP) pathway. PVN oxytocinergic neurons facilitate penile erection and sexual activity not only when activated by oxytocin itself but also by neurotransmitters such dopamine, excitatory amino acids, and NO; VGF peptides or hexarelin peptide analogues; drugs that increase NO concentration (NO donors); or the blockade of CB1 receptors in the PVN. Conversely, when oxytocinergic neurons are inhibited, for instance by GABA, opioid peptides/opiate drugs, or drugs that inhibit NOS activity, spontaneous (i.e., physiologically activated) or drug/neuropeptide-stimulated sexual activity is reduced. Appropriate references are cited throughout the manuscript.

The PVN (from which the main part of extra-hypothalamic oxytocinergic projections arise) (see above) was soon recognized as the area of the brain that is the most sensitive to the pro-erectile effect of oxytocin in male rats. Accordingly, doses as low as 3 picomoles were found to be able to induce penile erection (and yawning) when injected into this hypothalamic nucleus [87]. This is also true today, as oxytocin has also been recognized as being able to elicit penile erection after injection into other brain areas that contain the nerve endings of the oxytocinergic neurons arising from the PVN and its surrounding periventricular area and oxytocinergic receptors [19,20,88,89] (for a review [67,90]). In the PVN, oxytocin acts to induce penile erection by activating its own neurons (see Figure 2 and next section). Indeed, (i) oxytocin receptors have been identified in the PVN [91], (ii) oxytocin facilitates its own release in vitro and in vivo in the PVN [92,93,94] and excites its own neurons [95], and (iii) oxytocinergic nerve endings that impinge on the cell bodies of magnocellular oxytocinergic neurons have been identified in the PVN and in the SO of male rats [96]. Finally, electrolytic or chemical excitotoxic lesions of the PVN, which cause a complete depletion of oxytocin content in the brain and spinal cord, eliminates the pro-erectile effect of oxytocin and reduces drug-induced and noncontact penile erections in male rats (see [97,98]). Non-contact erections are penile erections seen in sexually potent male rats when they are put in the presence of an inaccessible receptive female rat that are caused by physiological and mainly olfactory (pheromones) sexual stimuli and are viewed as an index of sexual arousal [99,100,101]).

Results similar to those found after lesions of the PVN are seen when nonapeptide oxytocin receptor antagonists are injected into the PVN and/or into the lateral ventricles. Accordingly, these substances injected into the lateral ventricles of male rats in picomole amounts prevent penile erections induced by oxytocin and by other pro-erectile drugs and peptides (i.e., dopamine agonists, serotonin agonists, hexarelin peptide analogues, VGF-derived peptides, etc.) (see [64,65,66,67,85,102]) and also prevent noncontact erections [103].

Regarding the mechanism of oxytocin injected into the PVN, which activates its own neurons to induce penile erection, it is likely that the stimulation of oxytocin receptors causes an increase in the Ca^2+^ influx in the oxytocinergic cell bodies. This causes the activation of neuronal NO-synthase, the Ca^2+-^ calmodulin dependent enzyme that converts L-arginine in NO and citrulline, and that is present in the cell bodies of oxytocinergic neurons (see [104,105]), thereby increasing NO production. NO, in turn, activates the oxytocinergic neurons innervating extra-hypothalamic brain areas and the spinal cord through a cyclic guanosyne-monophosphate (cGMP)-independent and still unknown mechanism [106,107] (see also [64,65,66]). Accordingly, oxytocin-induced penile erection is not only abolished by d(CH_2_)_5_-Tyr(Me)^2^-Orn^8^-vasotocin, a potent and selective nonapeptide oxytocin receptor antagonist [108] given ICV, but also by omega-conotoxin GVIA, a potent inhibitor of voltage-dependent Ca^2+^ channels [109] that is injected in nanogram amounts into the PVN [110,111] and by neuronal NO synthase inhibitors, such as N(omega)-nitro-L-arginine methylester and S-methyl-thio-L-citrulline [112,113,114]. Conversely, the injection of drugs into the PVN that increase NO levels (so-called NO donors as nitroglycerin, sodium nitroprussiate, hydroxylamine, etc.) but not 8-Br-c-GMP levels, an active stable phosphodiesterase resistant analog of c-GMP, induce penile erections similar to those induced by oxytocin [112,115,116]. Finally, NO production increases in the PVN dialysate in parallel to penile erection in oxytocin-treated rats, and this increase is reduced by NO-synthase inhibitors injected into the PVN at doses that impair the erectile response of the neuropeptide [107]. Similar mechanisms occur in the PVN when penile erection takes place in physiological contexts, i.e., during noncontact erections and copulation or when oxytocinergic neurons are activated by other neurotransmitters or neuropeptides. Indeed, NO production increases in the PVN of sexually potent male rats when they show noncontact erection and during copulation as well [117]. In both cases the NO production is reduced by NO-synthase inhibitors injected into the PVN at doses that impair these behavioral responses [117]. In line with the facilitatory role of paraventricular NO in erectile function supported by the above findings, (i) impotent male rats have NO-synthase mRNA levels in the PVN that are only half of those found in sexually potent male rats [118], and (ii) the decrease in PVN NO-synthase mRNA levels is parallel to a decrease in PVN oxytocin mRNA levels and to an increase in opioid mRNA peptide levels, respectively [119] (see Section 3).

#### 2.1.2. PVN Oxytocinergic Neurons Are Also Activated by Other Neurotransmitters and Neuropeptides Present in the PVN Other Than by Oxytocin

Oxytocin is not the only compound that activates rat PVN oxytocinergic neurons projecting to extra-hypothalamic brain areas and that induces penile erection. In fact, many other compounds (neurotransmitters, neuropeptides and their agonists and sometimes also antagonists) have been identified to induce penile erection by acting on PVN oxytocinergic neurons. These include dopamine, glutamic acid, growth hormone (GH) secretagogue hexarelin-related peptides, and C-terminal VGF-derived peptides (Figure 2 and Table 2).

Dopamine activates oxytocinergic neurons and induces penile erection by acting on the dopamine receptors of the D2 family (D_2_, D_3_, and D_4_), which are all present in rat PVN oxytocinergic cell bodies, see [120,121], and are mainly of the D_2_ and D_4_ subtype [122,123,124,125], although some controversy exists on this point [126,127]. Glutamic acid activates oxytocinergic neurons and induces penile erection by acting mainly on N-Methyl-D-Aspartic acid (NMDA) receptors; however, a potential role of the α-amino-3-hydroxy-5-methyl-4-isoxazolepropionic acid (AMPA) receptors cannot be ruled out [106,128,129,130,131]. The exact mechanism of how GH secretagogue hexarelin-related peptides and VGF-derived peptides activate oxytocinergic neurons when injected into the PVN is still unknown. However, it is likely that these peptides act on specific receptors located in the oxytocinergic cell bodies that have yet to be identified and whose stimulation will lead to the activation of the oxytocinergic neurons mediating penile erection in the PVN. Accordingly, structure–activity relationship studies suggest that these receptors are different from those previously characterized that mediate GH release and feeding behavior [132,133,134]. Briefly, when activated, these receptors also induce penile erection by increasing Ca^2+^ influx in the cell bodies of oxytocinergic neurons, causing the activation of NO-synthase and an increase in NO production. This, in turn, activates oxytocinergic neurons with mechanisms similar to those described for dopamine and its agonists, oxytocin and NMDA [135]. In fact, hexarelin-related peptide-induced penile erection takes place concomitantly with increased NO production in the PVN and is reduced by the inhibition of PVN NO-synthase [135] and by ω-conotoxin, which blocks N-type voltage-dependent Ca^2+^ channels [133]. These results together with the ability of an hexarelin-related peptide analogue devoid of proerectile activity to prevent hexarelin-related peptide- but not apomorphine-, oxytocin- or NMDA-induced penile erection is in line with the existence of a specific receptor for hexarelin-related peptides in the cell bodies of PVN oxytocinergic neurons, the stimulation of which induces penile erection [136]. However, whether hexarelin-related peptides interfere with currently unidentified substances present in the PVN cannot be completely excluded. A similar mechanism is likely to occur with the C-terminal VGF-related peptides that cause penile erection when injected into the rat PVN. VGF is a protein that is expressed in neuroendocrine cells that is thought to play a role in the regulation of energy homeostasis and is proteolytically cleaved to yield multiple bioactive peptides (see [137]). Accordingly, (i) C-terminal VGF-immunoreactive fibres that impinge or pass close to oxytocinergic cell bodies have been identified in the rat PVN by double immunohistochemistry [138]; (ii) C-terminal VGF-related peptide-induced penile erection takes place concomitantly with increased PVN NO production [139]; and (iii) both penile erection and increased NO production are abolished by a blockade of oxytocinergic receptors created by d(CH_2_)_5_-Tyr(Me)^2^-Orn^8^-vasotocin injected into the lateral ventricles but not into the PVN, which is in line with the idea that C-terminal VGF-related peptides induce penile erection by activating central oxytocin neurotransmission [139]. In contrast to C-terminal VGF-derived peptides, neuroendocrine-regulatory peptide-1 (NERP-1), but not neuroendocrine regulatory peptide-2 (NERP-2), (both of which are VGF-derived peptides [140]), induces penile erection when injected into the lateral ventricles and into the arcuate nucleus but not into the PVN in male rats [141]. Since NERP-1-induced penile erection is not abolished by d(CH_2_)_5_-Tyr(Me)^2^-Orn^8^-vasotocin injected into the lateral ventricles, it is likely that this VGF-derived peptide induces penile erection with a mechanism that is not related to oxytocin [141]. Despite the above uncertainties, all of these compounds (dopamine, glutamic acid, hexarelin-related peptides, and C-terminal VGF-derived peptides) activate oxytocinergic neurons and induce penile erection with a mechanism similar to that of oxytocin, e.g., by activating neuronal NO synthase and by increasing NO content. This activates oxytocinergic neurons by projecting to the extra-hypothalamic brain areas and to the parts of the spinal cord that mediate penile erection by a c-GMP-independent and still unknown mechanism (see above). A detailed description of the role of PVN-oxytocinergic neurons in mediating penile erection induced by the above compounds can be found in earlier reviews [65,66,67,83].

#### 2.1.3. PVN Oxytocinergic Neurons Are Also Inhibited by Neurotransmitters and Neuropeptides Present in the PVN

PVN-oxytocinergic neurons projecting to extra-hypothalamic brain areas and to the spinal cord, whose activation induces penile erection, are also the target of other neurotransmitters and neuropeptides that inhibit rather than facilitate erectile function in rats. Among these, the best known are gamma-amminobutyric acid (GABA), opioid peptides, and endocannabinoids (Figure 2 and Table 2).

The inhibitory effect of GABA on the PVN-oxytocinergic neurons mediating penile erection has been ascertained by studies showing that muscimol, a GABA_A_ receptor agonist, injected into the PVN of male rats at doses that fail to induce gross behavioral alterations impairs penile erection caused not only by oxytocin, dopamine agonists, and N-methyl-D-aspartic acid (NMDA) [142] but also impairs noncontact penile erections [143]. In agreement with the hypothesis that muscimol inhibits penile erection by stimulating GABA_A_ receptors, bicuculline, a GABA_A_ receptor antagonist, abolishes the inhibitory effect of muscimol on erectile responses when injected into the PVN prior to muscimol, and baclofen, a GABA_B_ receptor agonist, is unable to induce penile erection when injected into the PVN [142,143]. GABA_A_ receptors, which control penile erection in an inhibitory manner, may be located in the cell bodies of oxytocinergic neurons. Accordingly, GABAergic nerve terminals impinge on the cell bodies of magnocellular and parvocellular oxytocinergic neurons in the rat PVN [144,145]. Due to the mechanism activated by the stimulation of the GABA_A_ receptor in the PVN to reduce/prevent the activation of oxytocinergic neurons, and hence penile erection, this seems to be related to the ability of these receptors when they are activated to inhibit the increase in NO production that usually occurs in the cell bodies of PVN oxytocinergic neurons when these are activated and when penile erection takes place. In fact, the inhibitory effect of muscimol that has been injected into the PVN on penile erection is parallel to a decrease in the increase of NO that takes place in the PVN when drug- and oxytocin-induced penile erection and noncontact erections occur, an effect prevented by the prior administration of bicuculline, which blocks GABA_A_ receptors, in the PVN [143,146].

Endogenous opioid peptides (i.e., enkephalins, endorphin, and dynorphin) [147,148] also exert an inhibitory effect on erectile function and sexual behavior. This is supported by the ability of morphine and other opiate drugs, which activate the various receptor subtypes (µ, κ and δ) of the endogenous opioid peptides, to inhibit penile erection and sexual behavior both in laboratory animals and in humans (see [68,147]). In fact, opiate addicts often complain about impotence and a decrease in sexual libido, and a usual sign of opiate abstinence is penile erection [149] (see [67,68] and references therein). Moreover, morphine, but not U-69,593, a potent stimulant of the κ opioid receptor subtype, prevents penile erection induced by oxytocin, apomorphine, and NMDA when injected into the PVN [150,151,152] (also see [68]). Since these inhibitory effects of morphine are antagonized by naloxone, a classical opioid receptor antagonist, these findings suggest that morphine inhibits oxytocin and drug-induced penile erection by stimulating opioid receptors of the µ subtype [150,151,152]. As found with muscimol (see above), the ability of morphine to prevent penile erection induced by oxytocin, dopamine agonists, NMDA, and also by SR 141716A (Rimonabant, [N-(piperidin-1-yl)-5-(4-chlorophenyl)-4-methyl-1H-pyrazole- 3-carboxyamide]), a selective antagonist of the CB1 cannabinoid receptor subtype [153] (see below), is secondary to the inhibition of the increase in NO production that takes place in the rat PVN after the injection of a dose of the above substances that induce penile erection [151,154].

Morphine injected into the PVN prevents penile erection induced by oxytocin, dopamine agonists, NMDA, and SR 141716A as well as noncontact penile erections and copulation [155]. This inhibitory effect occurs concomitantly to a decrease in the increase of NO production that take place in these physiological contexts [117,155]. The fact that endogenous opioid peptides and opiates inhibit penile erection and sexual behavior is also sustained by findings showing that impotent male rats (see also above) contain levels of opioid peptide messenger RNAs in the PVN that are higher than those found in sexually potent male rats and lower levels of oxytocin and NO-synthase messenger RNAs [118,119]. Together, these findings suggest that the stimulation of GABA_A_ receptors or opioid receptors inhibits penile erection by decreasing NO-synthase activity in the oxytocinergic neurons mediating penile erection. This causes a decrease in the release of oxytocin in extra-hypothalamic brain areas and in the spinal cord.

Endocannabinoids are the last neurotransmitters that may inhibit PVN oxytocinergic neurons mediating penile erection that have been identified so far. This became evident when SR 141716A, a selective antagonist of the CB1 receptor subtype [153], was found to be able to induce penile erection when injected into the PVN of male rats [156]. Apparently, the blockade of CB1 receptors in the PVN induces penile erection by activating oxytocinergic neurons with a mechanism that leads to the activation of glutamatergic neurotransmission in the PVN. Glutamic acid, in turn, activates oxytocinergic neurons by increasing Ca^2+^ influx into the cell bodies of these neurons. This activates NO production, which lead to the release of oxytocin in extra-hypothalamic brain areas and in the spinal cord. Accordingly, SR 141716A-induced penile erection takes place with an increase in extracellular glutamic acid and NO production in the PVN dialysate [154,157,158]. Penile erection and the increase in NO production but not in extracellular glutamic acid induced by SR 141716A are antagonized by the blockade of PVN-excitatory amino acid receptors of the NMDA subtype by (+)-MK-801 (dizolcipine, [(5R,10S)-(+)-5-methyl-10,11-dihydro-5Hdibenzo-[a,d]cyclohepten-5,10-imine hydrogen maleate]), a potent noncompetitive antagonist of excitatory amino acid receptors of the NMDA subtype [159], injected into the PVN and by the inhibition of NO synthase in the PVN [156,157,158]. In contrast to the blockade of PVN excitatory amino acid receptors and the inhibition of PVN NO synthase, the stimulation of the PVN opioid receptors (by morphine) antagonized not only penile erection and the increase in NO production but also the increase in extracellular glutamic acid induced by SR 141716A in the PVN dialysate [154].

Immunohistochemistry and autoradiography studies have shown that blocking the CB1 receptors blocked by means of SR 141716A, which leads to the increase in glutamic acid neurotransmission that activates the oxytocinergic neurons mediating penile erection, are located in the inhibitory GABAergic synapses that impinge on the glutamatergic synapses surrounding and impinging on oxytocinergic cell bodies [160]. However, due to the limitation of the immunohistochemistry technique, this study does not rule out the possibility that SR 141716A may also act on the inhibitory CB1 receptors located directly in the excitatory glutamatergic synapses impinging on oxytocinergic cell bodies (see [158]). Irrespective of the exact location of the CB1 receptors whose blockade leads to the increase in glutamatergic neurotransmission in the PVN, this study has also shown that chronic SR 141716A treatment induces a long lasting CB1-receptor increase in the rat PVN that is able to lead to a potentiated pro erectile effect of SR 141716A [160].

### 2.2. Ventral Tegmental Area

**Figure 3:**
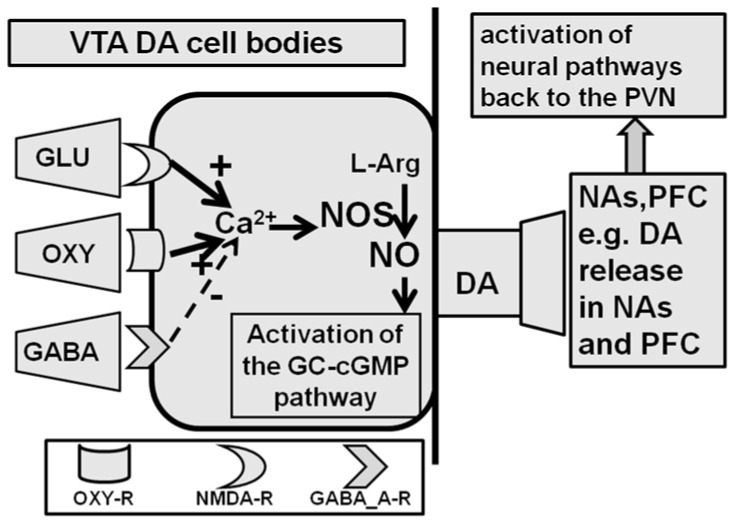
Oxytocin in the ventral tegmental area facilitates penile erection and copulatory activity in male rats. Briefly, oxytocin (OXY) acts on its own receptors (OXY-R) located in the cell bodies of mesolimbic/mesocortical dopaminergic (DA) neurons that originate in the ventral tegmental area (VTA) and project to the nucleus accumbens (NAs) and prefrontal cortex (PFC). This increases the influx of Ca^2+^ ions in the cell bodies of these DA neurons, causing the activation of nitric oxide synthase (NOS) found in these neurons, thus increasing NO production. NO, in turn, activates DA neurons through a guanylate cyclase (GC)-cGMP mechanism to release DA in the NAs and PFC, inducing the activation of yet unknown neural pathways that project back to the hypothalamic PVN, which contains the cell bodies of OXY neurons projecting to the spinal cord (SpC) that control penile erection and sexual behavior. DA neurons in the VTA may also be activated by glutamic acid (GLU) released (through the activation of NMDA receptors (NMDA-r) by synapses of GLU neurons originating in the ventral subiculum/amygdala or the PFC or inhibited by GABA through the activation of GABA_A_ receptors (see Figure 4). Details and references are reported in the Section 2.2.

The ventral tegmental area is another rat brain site where oxytocin induces penile erection. As recalled above, this brain area receives the nerve endings of the oxytocinergic neurons arising from the PVN and is reported be where the oxytocin receptors in rodents [161,162,163] and in humans [164,165,166] are. The idea that oxytocin induces penile erection when injected into this brain area was only discovered in 1997. More precisely, oxytocin induces penile erection when injected unilaterally into the caudal area but not into the rostral ventral tegmental area in a dose-dependent manner [88]. Higher doses of oxytocin must be injected into this area to induce penile erection when compared to those with it injected directly into the PVN (but close to those levels that cause penile erection when injected into the ventral subiculum of the hippocampus or into the posteromedial cortical nucleus of the amygdala, see below). Briefly, oxytocin injected in the ventral tegmental area of male rats induces penile erection by activating mesolimbic dopaminergic neurons projecting from the ventral tegmental area to the nucleus accumbens’ shell. This leads to the activation of yet unknown neural pathways running to the incerto-hypothalamic dopaminergic neurons that impinge on paraventricular oxytocinergic neurons [88,167]. In line with this hypothesis, the available evidence suggests that oxytocin acts on the oxytocinergic receptors located in the cell bodies of mesolimbic dopaminergic neurons. This increases Ca^2+^ influx into the cell bodies of these neurons, leading to the activation of neuronal NO-synthase [168], a mechanism similar to that reported for the PVN (see above). However, in contrast to the cell bodies of PVN-oxytocinergic neurons (Figure 3, see the Section 2 and Section 2.1.1), NO in the cell bodies of dopaminergic neurons activates guanylate cyclase, thereby increasing the concentration of c-GMP. Accordingly, d(CH_2_)_5_-Tyr(Me)^2^-Orn^8^-vasotocin, a potent oxytocin receptor antagonist, S-methyl-thio-L-citrulline, a potent inhibitor of neuronal NO-synthase, and ODQ (1H-[1,2,4]oxadiazole[4,3-a]quinoxalin-1-one), a soluble guanylate cyclase inhibitor [169], injected into the rat caudal ventral tegmental area before oxytocin, all eliminate penile erection and the concomitant increase in extra-cellular dopamine in the nucleus accumbens’ shell induced by oxytocin [88]. Likewise, 8-bromo-c-GMP, an active phosphodiesterase-resistant c-GMP analogue that does not induce penile erection when injected into the PVN (see above), causes penile erection when injected into the caudal ventral tegmental area and increases extra-cellular dopamine in the nucleus accumbens’ shell, as found in the case of oxytocin injected into the caudal ventral tegmental area [90,167,168]. In line with this mechanism, (i) the blockade of D2 receptors by haloperidol injected into the nucleus accumbens’ shell impairs penile erection induced by oxytocin injected into the ventral tegmental area [88], and (ii) double immuno-fluorescence experiments show that in the ventral tegmental area, oxytocin fibres make contact with the cell bodies of dopaminergic neurons, which had previously been labeled with the retrograde tracer Fluorogold injected into the nucleus accumbens’ shell [88,168]. A facilitatory role of the NO-cGMP signalling system in the control of penile erection at the level of the ventral tegmental area is also suggested by the ability of phosphodiesterase inhibitors clinically used for the therapy of erectile dysfunction, i.e., sildenafil and vardenafil, to increase the number of noncontact erections when injected directly into the ventral tegmental area of male rats, a response that takes place concomitantly with an increase of extra-cellular dopamine in the nucleus accumbens’s shell dialysate [170].

How the activation of mesolimbic dopaminergic neurons and of dopamine receptors in the nucleus accumbens (and medial prefrontal cortex) induced by oxytocin injected into the ventral tegmental area leads to penile erection is still unknown. One possibility is that such activation leads to the stimulation of the activity of neural pathways that have yet to be identified that increase the activity of incerto-hypothalamic dopaminergic neurons and the release of dopamine in the PVN, thereby activating oxytocinergic neurons projecting to the spinal cord and mediating penile erection (see above and [88,168,171]). In line with this hypothesis, (i) oxytocin injected into the caudal ventral tegmental area at a dose that induces penile erection increased extra-cellular dopamine in the dialysate obtained from the nucleus accumbens and from the PVN [171], and (ii) these effects are eliminated by the prior injection of the oxytocin receptor antagonist d(CH_2_)_5_-Tyr(Me)^2^-Orn^8^-vasotocin into the caudal ventral tegmental area or by the injection of haloperidol into the nucleus accumbens [88,171]. These findings revealed the presence of an important interaction in the ventral tegmental area between the synapses of the oxytocinergic neurons originating in the PVN and the cell bodies of mesocorticolimbic dopaminergic neurons, which reach the nucleus accumbens (and the medial prefrontal cortex). This led to the suggestion that this oxytocin–dopamine interaction in the ventral tegmental area may be the basis for the connection between the mechanisms regulating the anticipatory (sexual arousal, motivation, and reward) and consummatory phases (erectile function and copulation, sexual performance) of sexual behavior [90] (see Section 4). Interestingly recent studies based on the use of novel experimental designs have confirmed not only the existence of oxytocinergic receptors in mesolimbic dopaminergic cell bodies in the mouse ventral tegmental area [172], but also that the release of oxytocin in this brain area is a key factor in the eliciting the social rewards generated by social interactions, which is critical for promoting prosocial behaviors [22]. This was ascertained by the selective activation or inhibition of the oxytocinergic neurons that originate in the PVN and project to the cell bodies of mesocorticolimbic dopaminergic neurons in the ventral tegmental area coupled to behavioral experiments aimed at measuring social rewards (conditioned place preference) in various lines of knock-in and knock-out mice with genetically modified paraventricular oxytocin neurons [22].

### 2.3. Hippocampus

The hippocampus is a complex brain area that is divided into roughly two main partitions, the dorsal and the ventral hippocampus, which differ in function (see [173] and references therein). The CA1 field of the dorsal hippocampus was the other area of the rat brain containing oxytocinergic fibres and receptors (see [14,15]) identified in 1985 in which the injection of oxytocin induced penile erection [87]. However, in contrast to the PVN, in the CA1 field, oxytocin was only able to induce penile erection when injected bilaterally and at doses higher than those active in the PVN [87,174]. Nonetheless, the idea that oxytocin in the hippocampus plays a role in the induction of penile erection is also supported by other findings. Accordingly, (i) apomorphine, a dopamine agonist that induces penile erection by activating PVN oxytocinergic neurons [65,66], increases oxytocin content in the hippocampus [175]; (ii) lesions of the ventral hippocampus (which is functionally connected to the CA1 field, see [176]), or of the medial amygdala or the medial septum, which decrease oxytocin content in the hippocampus [177], impairs penile erection in rats [177,178], and L-368,899 [1-(((7,7-dimethyl-2(S)-(2(S)-amino- 4-(methylsulfonyl) butyramido) bicyclo[2.2.1]-heptan- 1(S)-yl)methyl) sulfonyl) -4-(2-methylphenyl)piperazine], a non-peptide oxytocin receptor antagonist that accumulates in the hypothalamus, the amygdala, and the ventral hippocampus impairs male sexual behavior in rhesus monkeys when given systemically [179]. In earlier studies, injections of oxytocin into the subiculum of male rats were found to be inactive on penile erection [86]. However, recent and more accurate microinjection studies have allowed the identification of a region of the ventral subiculum where unilaterally injected oxytocin was able to induce penile erection in a dose-dependent manner [89] and at doses similar to those found active when injected unilaterally into the caudal part of the ventral tegmental area [88]. Why oxytocin has to be injected bilaterally into the CA1 field of the dorsal hippocampus while unilateral injections of the neuropeptide in the subiculum of the ventral hippocampus are already able to induce penile erection in male rats is unknown. However, this might reflect the specific connections and functional roles of the two partitions of the hippocampus recalled above. The mechanism by which oxytocin induces penile erection when injected into the CA1 field of the dorsal hippocampus is also unknown [86].

**Figure 4:**
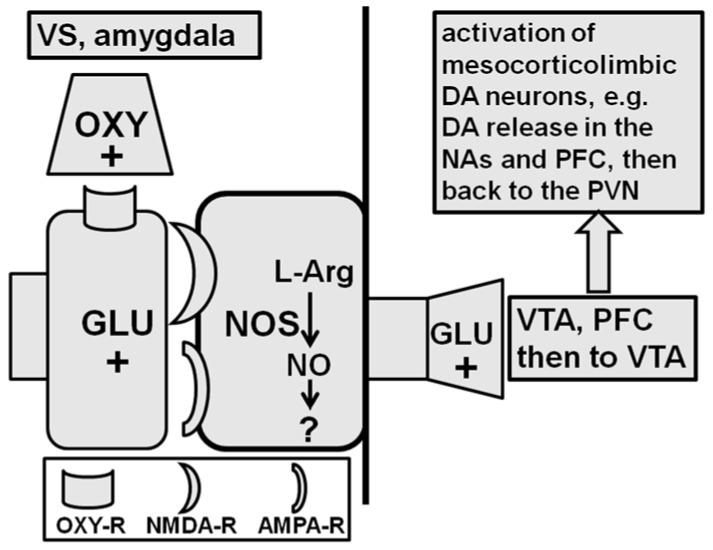
Oxytocin in the ventral subiculum (VS) and/or the amygdala (posteromedial nucleus) facilitates penile erection and copulatory activity in male rats. Briefly, oxytocin (OXY) acts on receptors (OXY-R) located in the cell bodies of possibly glutamatergic (GLU) neurons containing NO synthase (NOS), thus increasing NO production in these two brain areas. This leads to the activation of GLU neurons projecting directly or indirectly (through the PFC and then from there to the VTA) to the cell bodies of mesolimbic/mesocortical dopaminergic (DA) neurons that originate in the VTA and project to the n. accumbens (NAs) and prefrontal cortex (PFC). GLU in the VTA acts on NMDA receptors (NMDA-R) located in the cell bodies of DA neurons, although a role of AMPA receptors (AMPA-R) cannot be completely excluded, possibly through the activation of the guanylate cyclase-cyclic GMP pathway. This causes the release of DA in the NAs and PFC, leading to the activation of yet unknown neural pathways that project back to the PVN, as described for OXY acting in the VTA (see Figure 3). Details and references are reported Section 2.3 and Section 2.4.

As for the ventral subiculum, the available data support the hypothesis that oxytocin injected into this area induces penile erection by acting on the oxytocinergic receptors located in neurons containing neuronal NO-synthase, causing an increase in NO production. By acting as an intra- or intercellular messenger, NO activates glutamic acid neurotransmission. This induces penile erection, possibly through the activation of glutamatergic efferent projections from the ventral subiculum to the extra-hippocampal brain areas that modulate the activity of mesolimbic dopaminergic neurons (i.e., the ventral tegmental area, the prefrontal cortex, the PVN, and possibly the bed nucleus of the stria terminalis) (Figure 4, see below and [23,24,88,89,168,180,181]). Accordingly, intracerebral microdialysis experiments show that in male rats, oxytocin injected into the ventral subiculum at doses that induce penile erection increases NO production and extra-cellular glutamic acid in the dialysate obtained from the ventral subiculum [180] and of extra-cellular dopamine in the nucleus accumbens and medial prefrontal cortex [180,181]. These responses were antagonized by the oxytocin receptor antagonist d(CH_2_)_5_-Tyr(Me)^2^-Orn^8^-vasotocin, the neuronal NO-synthase inhibitor S-methyl-thio-L-citrulline, and the NO scavenger haemoglobin when injected into the ventral subiculum a few minutes before oxytocin was [180]. Finally, in agreement with the mechanism of action proposed above, the activation of glutamatergic neurotransmission by NMDA injected into the ventral subiculum induces penile erection [180].

The type of ventro-subicular efferent projections, which lead to the activation of mesolimbic and mesocortical dopaminergic neurons and the increase of extra-cellular dopamine in the nucleus accumbens and the medial prefrontal cortex dialysate, is unknown at present. However, these projections cause the activation of glutamatergic neurotransmission in the ventral tegmental area, which activates the mesolimbic and mesocortical dopaminergic neurons that project to the nucleus accumbens and to the medial prefrontal cortex, respectively. Accordingly, penile erection induced by oxytocin injected into the ventral subiculum of male rats (i) takes place concomitantly due to an increase of extra-cellular glutamic acid in the ventral tegmental area but not in the nucleus accumbens or medial prefrontal cortex dialysate and (ii) is antagonized by (+)MK-801 injected into the ventral tegmental area but not into the nucleus accumbens [89,181]. Whether the increased concentration of glutamic acid found in the ventral tegmental area dialysate after oxytocin injection into the ventral subiculum is due to an increased release of the excitatory amino acid from the neurons originating in the subiculum (e.g., projecting directly to the ventral tegmental area) or in other brain areas (e.g., the prefrontal cortex and/or the nucleus accumbens itself) is unknown. Nonetheless, this increase in glutamic acid activates mesolimbic and mesocortical dopaminergic neurons and increases the release of dopamine in the nucleus accumbens and probably in the medial prefrontal cortex. The activation of the dopamine receptors in these two areas causes the activation of the incerto-hypothalamic dopaminergic neurons. This increases the release of dopamine in the PVN, thereby activating the oxytocinergic neurons that project to the spinal cord inducing penile erection (see above and [88,89,167,179,181]).

### 2.4. Amygdala (Posteromedial Cortical Nucleus)

The amygdala is another brain area that receives oxytocin fibres from the PVN and contains oxytocin receptors (see [161,163,182]). This almond-shaped structure comprises approximately 13 nuclei, which are further subdivided into extensive internuclear and intranuclear connections. These nuclei are functionally organized in five major groups: basolateral nuclei, cortical-like nuclei, central nuclei, other amygdaloid nuclei, and extended amygdala [183]. A large amount of reports initiated at the end of 2000 and that are still ongoing regarding the intranasal administration of oxytocin support the hypothesis that oxytocin in the amygdala is involved in numerous central functions, i.e., anxiolysis, social memory and cognition, socially reinforced learning, empathy, face processing and fear in humans, autism, mental pathologies (schizophrenia, depression, drug dependence), erectile function, and sexual behavior (see [33,184,185,186,187,188,189,190,191,192,193,194,195]). However, the idea that oxytocin induces penile erection when injected into the posteromedial cortical nucleus of the amygdala of male rats was only discovered in 2009 [89]. Interestingly, this nucleus is sexually dimorphic in rats and has been reported to play a role in reproductive behavior [196,197] in non-contact erections and in the regulation of penile erections that occur in other contexts [178]. It was soon discovered that penile erection induced by oxytocin injected into the posteromedial cortical nucleus of the amygdala was parallel to an increase in extra-cellular dopamine in the nucleus accumbens’ shell dialysate, as found in the ventral subiculum [89]. How the injection of oxytocin into this nucleus of the amygdala induces penile erection in rats is unknown. The available data show that penile erection and the increase in extra-cellular dopamine in the nucleus accumbens are mediated by the stimulation of oxytocinergic receptors, with these effects being eliminated when d(CH_2_)_5_-Tyr(Me)^2^-Orn^8^-vasotocin is injected into this nucleus of the amygdala before oxytocin [89]. Irrespective of the mechanism responsible for the oxytocin effect in this nucleus of the amygdala, penile erection induced by the neuropeptide is abolished by cis-flupenthixol, a potent antagonist of all D1 and D2 dopamine receptors, injected into the shell of the nucleus accumbens and by (+)MK-801, which blocks NMDA receptors, when injected into the ventral tegmental area but not into the nucleus accumbens, similar to what was found when oxytocin was injected into the ventral subiculum [89]. Thus, it is likely that the injection of oxytocin into this nucleus of the amygdala activates glutamic acid neurotransmission in the ventral tegmental area. This activates mesolimbic dopaminergic neurons, inducing penile erection (see Figure 4). As several studies show that neural pathways interconnect this nucleus with the ventral subiculum in rats [198,199], it is likely that these two areas interact each other, even if direct pathways from the amygdala to the nucleus accumbens or ventral tegmental area have been also identified [200,201]. Further studies are required to verify this possibility.

### 2.5. Bed Nucleus of the Stria Terminalis

**Figure 5:**
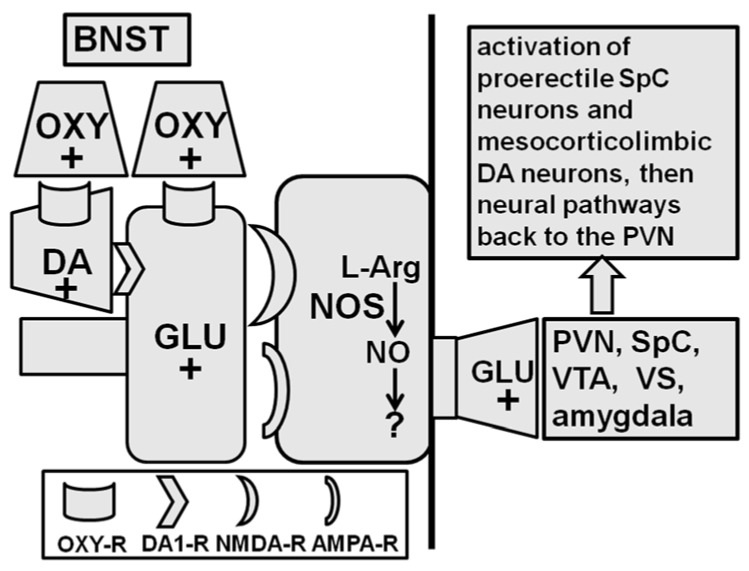
Oxytocin in the bed nucleus of the stria terminalis (BNST) facilitates penile erection and copulatory activity in male rats. Briefly, oxytocin (OXY), by acting on receptors (OXY-R) located in the nerve endings of glutamatergic (GLU) neurons, increases GLU release from these synapses, which impinge on cell bodies of GLU neurons containing NO synthase (NOS). Here, GLU acts on NMDA (NMDA-R) and AMPA receptors (AMPA-R) and activates NOS, increasing NO production and activating the GLU neurons that project directly or indirectly to the hypothalamic (PVN) and extra-hypothalamic brain areas such as the ventral tegmental area (VTA), ventral subiculum (VS), amygdala, and spinal cord (SpC), activating mechanisms leading to penile erection and sexual behavior. Microdialysis experiments support the hypothesis that OXY in the BNST also acts on OXY receptors located on the nerve terminals of dopaminergic (DA) neurons that originate in the VTA and impinge on the OXY-R-bearing GLU nerve endings, thus contributing to facilitating GLU release. Details and references are reported in the Section 2.5.

The bed nucleus of the stria terminalis is the last brain area discovered so far where the injection of oxytocin has been found to able to induce penile erection (and yawning) in male rats [23,24] (Figure 5). When injected into this brain area, the minimal effective dose of oxytocin was 20 ng, while the maximal response was seen with 100 ng. Additionally, in the bed nucleus of the stria terminalis, the pro-erectile effect of oxytocin is mediated by oxytocin receptors, as it is antagonized by d(CH_2_)_5_Tyr(Me)^2^-Orn^8^-vasotocin when it is injected into the bed nucleus before oxytocin, and when injected into the bed nucleus at the dose of 100 ng, Arg^8^-vasopressin is unable to induce penile erection. Neuropharmacological and microinjection studies first [23] and microdialysis and immunohistochemical experiments later [24] revealed that oxytocin injected into the bed nucleus induces penile erection (and yawning) by increasing both glutamatergic (and nitrergic) and dopaminergic neurotransmission in the bed nucleus. Briefly, microinjection studies first revealed that oxytocin injected into the bed nucleus increases the release of glutamic acid from the glutamatergic nerve endings of neurons possibly originating in the ventral subiculum of the hippocampus and/or the amygdala, which impinge on the NO synthase-containing cell bodies of the glutamatergic neurons projecting to the PVN and/or medial preoptic area, ventral tegmental area, ventral subiculum, and/or amygdala, activating the neural pathways controlling penile erection as previously described (Figure 5, see Section 2 and Section 2.5). The microinjection experiments also ruled out the involvement of other neurotransmitters (e.g., GABA) and neuropeptides (e.g., corticotrophin releasing factor) in the pro-erectile effect of oxytocin injected in the bed nucleus. Subsequent microdialysis studies showed that oxytocin injected into the bed nucleus not only activates the release of glutamic acid but also the release of dopamine from the nerve terminals of the dopaminergic neurons that originate in the ventral tegmental area and impinge on the same glutamatergic nerve endings containing the oxytocinergic receptors on which oxytocin acts to release glutamic acid. Dopamine released by oxytocin by acting on D1 but not D2 receptors stimulates the same NO synthase-containing cell bodies of the glutamatergic neurons projecting to the PVN and/or the medial preoptic area, which activate the neural pathways mediating penile erection that are present in these brain areas. In line with this possibility, dopamine D1 receptor and excitatory amino acid receptor blockade in the bed nucleus of the stria terminalis by SCH-23390 [R(+)-7-chloro-8-hydroxy-3-methyl-1-phenyl-2,3,4,5-tetrahydro- 1H-3-benzazepine hydrochloride)], a selective and potent D1 receptor antagonist, or by (+) MK-801 or 6-cyano-7-nitroquinoxaline-2,3-dione disodium salt (CNQX), two antagonists of excitatory amino acid receptors of the NMDA and AMPA subtype, respectively, reduce/abolish penile erection induced by oxytocin injected into the bed nucleus [23]. Moreover, double labelling immunohistochemical experiments revealed oxytocin-positive neuronal structures close to tyrosine hydroxylase-positive (dopaminergic) neurons or NO synthase-positive cell bodies surrounded by intense vesicular glutamate transporter 1-stained (glutamatergic) synapses in the bed nucleus sections in which oxytocin injections induce penile erection (and yawning) [24]. As discussed below (see Section 4) these results confirm a role of the bed nucleus of the stria terminalis in a complex circuitry controlling both the consummatory (penile erection and sexual performance) and the anticipatory (sexual arousal and sexual motivation) phases of sexual behavior through the dopaminergic, glutamatergic, and oxytocinergic pathways that reciprocally interconnect many of the brain areas recalled above [67,90].

### 2.6. Spinal Cord

The spinal cord is the other area of the central nervous system in which oxytocinergic fibres and receptors are found [161,182] and in which oxytocin is thought to act to facilitate penile erection in male rats [8,9,80,202]. Accordingly, spinal oxytocinergic fibres originating in the PVN participate in the descending pathways that control the spinal autonomic neurons innervating the erectile tissues of the genital apparatus (Figure 5). Oxytocinergic fibres make synaptic contacts in the preganglionic sympathetic and parasympathetic cell columns of the dorsal horns at the thoraco-lumbar and lumbo-sacral tract levels, with the spinal neurons innervating penile cavernous corpora [80,202,203]. Synaptic contacts were identified by the labeling of the spinal neurons that originate in the penis and that reach the spinal cord using retrograde tracers injected into the cavernous corpora in combination with double immuno-fluorescence and confocal laser microscopy [8,9]. In agreement with the above studies, in anaesthetized male rats, intrathecal oxytocin given in cumulative doses in the lumbo-sacral but not thoraco-lumbar level causes dose-dependent intracavernous pressure increases. The oxytocin response was eliminated by d(CH_2_)_5_-Tyr(Me)^2^-Orn^8^-vasotocin and by the sectioning of pelvic nerves [80,202]. Together, these findings demonstrate that oxytocin injected into the lumbo-sacral spinal cord increases intracavernous pressure. They also suggest that oxytocin is released when the PVN is activated in a physiological context, which may be a potent spinal pro-erectile neuron stimulant that projects to the cavernous corpora. In line with the existence of neuronal pathways that interconnect the PVN and the spinal nerves innervating the cavernous corpora, electrophysiological studies in anaesthetized male rats have shown that the electrical stimulation of the dorsal nerve of the penis activates oxytocinergic neurons in the PVN [204,205,206] and that the stimulation of the PVN as well as the stimulation of the medial preoptic area induces intracavernosal pressure increases [207,208,209,210]. However, it is not clear whether oxytocin directly or indirectly activates these spinal pro-erectile neurons. In this regard, it is noteworthy that the pro-erectile spinal neurons, which are activated by oxytocin to induce its pro-erectile effect, are also synaptically contacted by the serotoninergic neurons that originate in the nucleus paragigantocellularis of the reticular formation of the medulla oblongata [8,211]. The elimination of these serotoninergic neurons facilitates ejaculation and penile reflexes in male rats [212,213].

**Figure 6:**
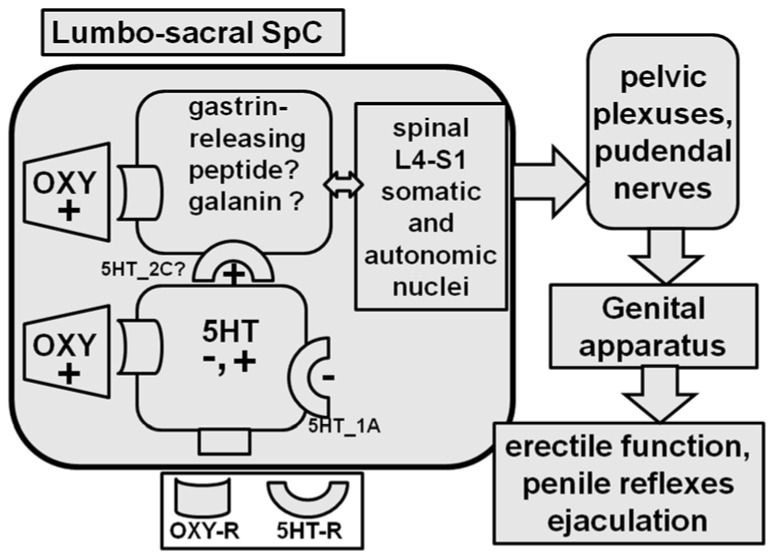
Oxytocin in the lumbosacral spinal cord facilitates erectile function and ejaculation in male rats. Briefly, oxytocin (OXY), by acting on receptors (OXY-R) located in the lumbo-sacral L4-S1 spinal cord, activates pro-erectile (gastrin-releasing peptide containing?) and proejaculatory (galanin-containing?) neurons projecting to the spinal somatic and autonomic nuclei, which send their neural projections through the pelvic nerves to the pelvic plexuses and pudendal nerves to the genital apparatus in order to control erectile function (including penile reflexes) and ejaculation. In the same lumbo-spinal tract, oxytocin may also influence erectile function and ejaculation by controlling serotonin (5HT) release from 5-HT neurons originating in the nucleus paragigantocellularis of the reticular formation. The neurotransmitter 5-HT is another neurotransmitter that plays a role in erectile function and ejaculation at spinal level. Details and references are reported in the Section 2.6.

Since 5HT2C receptor agonists induce penile erection in male rats when injected intracerebroventricularly, but not into the PVN, and since 5HT_2C_ receptor antagonists also impair penile erection induced by dopamine agonists and oxytocin, while dopamine antagonists do not abolish penile erection induced by 5HT_2C_ agonists (see [214] and references therein), it has been suggested that oxytocin not only acts directly on spinal pro-erectile neurons but also increases the pro-erectile effect of 5HT_2C_ receptors in the lumbo-sacral spinal cord [214] (Figure 6). This led the suggestion that the activation of the 5HT_2C_ receptors located in the spinal cord down-stream to those of dopamine and oxytocin is a common mechanism of the pro-erectile effect of these substances [214] and even of ACTH-MSH peptides [215]. However, oxytocin might also facilitate the activity of spinal descending serotoninergic neurons acting in the nucleus paragigantocellularis, where the cell bodies of these neurons are located. Accordingly, oxytocinergic neurons that originate in the PVN and that reach the nucleus paragigantocellularis have been identified in male rats [216]. Conversely, whether oxytocin is involved in the pro-ejaculatory effects of drugs that activate 5HT_1A_ receptors (i.e., 8-OH-DPAT) is still controversial. Although these drugs reduce mount and intromission frequency and ejaculation latency [217,218] and increase plasma oxytocin levels when given systemically [219,220], they are unable to induce penile erection [214] or affect sexual behavior in sexually active male rats when injected into the PVN [217]. Nevertheless, a partial reduction of these pro-ejaculatory effects of 8-OH-DPAT was obtained in male rats treated with an oxytocin receptor antagonist given ICV [215].

Oxytocin neurons that originate in the PVN and that project to the lumbosacral spinal cord are also important for ejaculation [221]. Accordingly, this sexual response is controlled by the so-called spinal cord ejaculation generator, which contains a population of lumbar spinothalamic neurons that co-express galanin, gastrin-releasing peptide, cholecystokinin, and enkefalin, whose release controls ejaculation and also penile erection by releasing these peptides in the lumbar and sacral autonomic and motor nuclei [222,223,224] (Figure 6). Since the selective destruction of galanin-containing neurons has been reported to eliminate ejaculation but not penile erection in male rats, this led to the suggestion that galanin neurons are the spinal cord ejaculation generator [221]. Interestingly, gastrin-releasing peptide containing neurons express oxytocinergic receptors and send their axons to the sacral autonomic nucleus and to the somatic spinal nucleus in the lower lumbar (L5-L6) and the upper sacral (S1) spinal cord, which innervates the bulbocavernous and ischiocavernous striated muscles attached at the base of the penis [225,226]. Sophisticated electrophysiological experiments have recently shown that these neurons are activated not only by the oxytocin released by the neurons originating in the PVN but also by optogenetic stimulation in adult male OXTR promoter-human heparin-binding epidermal growth factor-like growth factor (human diphtheria toxin receptor; Dxtr)-channel rhodopsin (ChR2)-EYFP BAC (Oxtr-ChR2-EYFP) transgenic rats [224]. The same group also found that oxytocin influences male sexual activity via not only synaptic but also non-synaptic release in the spinal cord; that is, oxytocin neurons can release oxytocin by exocitosis from axonal varicosities, allowing the neuropeptide to act by means of diffusion—a sort of localized volume transmission—to reach its own receptors in the lumbar spinal cord [227]. In line with the possible role of oxytocin at the spinal cord level in the control of ejaculation, a few orally acting nonpeptide oxytocin receptor antagonists have been developed in recent years in order to test therapies for premature ejaculation [228,229]. However, in spite of the above preclinical studies, which support a role of spinal oxytocin in ejaculation, recent studies failed to demonstrate a meliorative effect of cligosiban, an orally administered oxytocin receptor antagonist being developed to treat premature ejaculation in men with lifelong premature ejaculation in a randomized, double-blind, placebo-controlled phase IIb trial [230] against the results of another randomized, double-blind, placebo-controlled proof-of-concept trial study [231]. Further studies are necessary to ascertain if oxytocin antagonists may be used in the treatment of this sexual dysfunction.

## 3. Oxytocin and Sexual Behavior

It is well known that sexual behavior has a key role in the reproduction of all living animals, from insects to mammals, humans included. In mammals, sexual behavior is arranged into anticipatory and consummatory phases, and different quantifiable and reproducible parameters have been defined in these two phases in both males and females in a large number of studies from 1960 to present. The majority of the studies on sexual behavior have been completed in rats due to their availability, and well-defined sequence of copulatory behavior and its parameters (i.e., the number of mounts and intromissions of the penis into the vagina in a series of copulatory activity starting with a mount and ending with ejaculation, and the post ejaculatory interval, the time interval between an ejaculation and the beginning of a new series of copulatory activity) in the male (see [72,73,74,232,233,234]) and of the well-defined proceptive and receptive behavior in the female, the first being characterized by the first by darts, hops, and ear wiggling episodes, and the second of which being defined by lordosis (e.g., arching of the female back when the male mounts the female from back and touches her flanks and deflection of the tail to one side) (see [235,236]). However, extensive data on sexual behavior are also available for other mammals, i.e., mice (see [237,238]), hamsters [239], prairie and montane voles (see [240]), monkeys [241], and other animal species as well, i.e., the Japanese quail (see [242,243]) and the zebra finch (see [244]).

Penile erection followed by mounts and intromissions, seminal emission, and ejaculation define the consummatory phase of the male sexual response, while vaginal lubrication, clitoris erection, lordosis (which does not occur in women), and orgasm define the female sexual response. These responses are preceded by an anticipatory/appetitive phase, which comprises motivation towards and the search for an appropriate partner for copulation (see [72,73]). Briefly, when visual, auditory, olfactory, tactile, and also imaginative (in humans) sexual stimuli reach the central nervous system high centres, this activates the still unknown neural pathways that drive sexual information from the brain through the spinal cord and autonomous nervous system to the genital apparatus. This induces penile erection in males and vaginal lubrication/clitoris erection in females, making sexual intercourse that will culminate with ejaculation and orgasm feasible (see [64,73,75,76,245,246,247] and references therein) (see Figure 1 for a simplified representation of central and peripheral neural pathways that control erectile function and sexual behavior in males and females).

Male and female sexual behavior is also highly dependent on sexual hormones, e.g., testosterone in males and estrogen and progesterone in females, which are produced and released by the sexual glands (testes and ovaries), which are under the control of the hypothalamic–pituitary–gonadal (HPG) axis (see [72,73,248,249,250]). Briefly, the hypothalamus releases the gonadotropin-releasing hormone (GnRH), which activates the pituitary gonadotrophic cells to release the follicle stimulating hormone (FSH) and luteinizing hormone (LH) into the blood circulation. FSH and LH, in turn, stimulate the testes to release testosterone and the ovaries to release estrogen and progesterone. In laboratory animals, the removal of the testes, which eliminates testosterone in males, and of the ovaries, which eliminates the cyclic raises of estrogen and progesterone, which are responsible of the estrous cycle in females, rapidly abolishes sexual behavior in both sexes. This can usually be restored by the appropriate administration of the missing sexual hormones in both males and females.

### 3.1. Oxytocin and Male Sexual Behavior in Laboratory Animals

**Figure 7:**
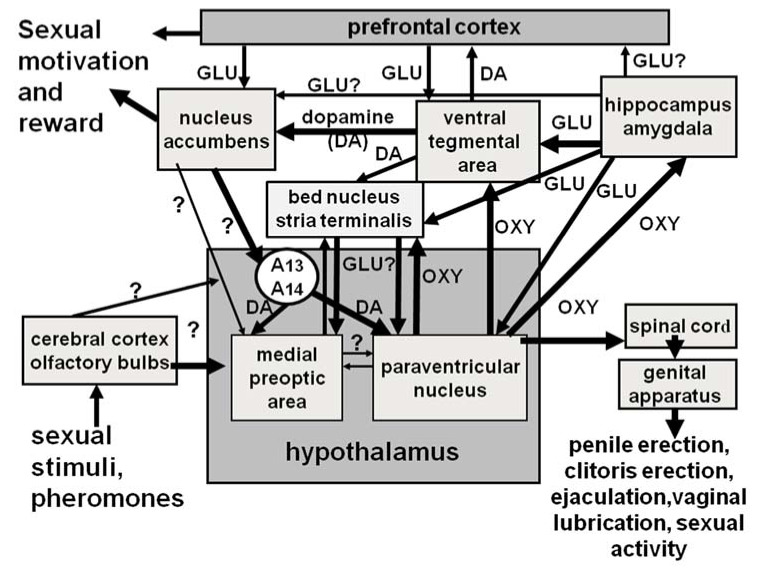
Oxytocin (OXY) participates in a complex neural circuit that control and influences sexual motivation and reward and sexual performance at the central level together with other neurotransmitters and/or neuropeptides. Briefly, oxytocinergic neurons that originate in the PVN send their projections to spinal cord and extra-hypothalamic brain areas, i.e., ventral tegmental area, hippocampus, amygdala, bed nucleus of the stria terminalis, and other brain areas. When activated by physiological stimuli (e.g., pheromones produced by a sexually receptive female) or by neurotransmitters (i.e., dopamine (DA), glutamic acid (GLU), and others), oxytocinergic neurons that project to the spinal cord improve penile erection and sexual performance, while those that project to the ventral tegmental area, hippocampus, amygdala, and bed nucleus of the stria terminalis directly or indirectly increase the activity of the mesolimbic and mesocortical dopaminergic neurons. The activation of these neurons, which have their cell bodies in the ventral tegmental areas and project to the nucleus accumbens and medial prefrontal cortex, allows, in turn, a modulation of sexual motivation and reward. Dopamine released in the accumbens and medial prefrontal cortex not only modulates sexual motivation and reward but also activates currently unknown neural pathways that improve the activity of incerto-hypothalamic dopaminergic neurons, which originated from the A13-A14 groups of Dahlstrom and Fuxe, and impinge on PVN oxytocinergic neurons that project to the spinal cord, causing penile erection and sexual activity. The activity of this circuit can be also modified by activation of (i) oxytocin acting directly in the ventral tegmental area or indirectly in the hippocampus (ventral subiculum), amygdala, and/or bed nucleus of the stria terminalis (which all receive oxytocinergic projections from the PVN) or (ii) by glutamatergic (GLU) neurons that project from these brain areas to the ventral tegmental area directly or indirectly (through the prefrontal cortex), causing a modulation of both sexual motivation/reward and erectile function/sexual activity, as described above (appropriate references are cited in the manuscript and in the References section).

Several neurotransmitters and neuropeptides play a role at the central and peripheral levels in the anticipatory and consummatory phases of sexual behavior. Among neuropeptides, the most studied are oxytocin, adrenocorticotropin (ACTH), α-melanocyte stimulating hormone (α-MSH), and opioid peptides (see [68,147,251,252,253,254]), although other peptides also are known to be involved (for a review, see [67] and references therein). While all of the above neuropeptides induce their effect on sexual behavior by acting in the hypothalamus and its nuclei (e.g., lateral hypothalamus, PVN, ventromedial nucleus and arcuate nuclei) and/or the medial preoptic area, oxytocin also acts in other brain areas (i.e., ventral tegmental area, hippocampus, amygdala, bed nucleus of the stria terminalis, medulla oblongata, and spinal cord), in which it usually interacts in a coordinated way with neurotransmitters such as dopamine, glutamic acid, GABA, and NO to control sexual performance, sexual motivation, or arousal across different brain areas (see Table 3), which are part of a complex neural circuit mediating sexual behavior and its main phases, as discussed in detail in Section 4 (Figure 7) (see [23,24,65,66,67,85,90,120,121]).

As recalled above, an enhancing effect of oxytocin on sexual behavior was first described in 1963 when intravenous oxytocin was reported to be able to shorten the latency to the first ejaculation and to retard the sexual exhaustion of male rabbits paired with receptive female rabbits [53]. Such a facilitatory role was confirmed in the 1980s, when oxytocin was found to be able to facilitate copulatory behavior in sexually potent male rats [60,255,256], in male mice [257], in aged male rats [258], and in dominant but not in subordinate male squirrel monkeys [259]. In sexually experienced male rats, the main effects of oxytocin administered either intraperitoneally (200 ng/rat) or intracerebroventricularly (1 ng/rat) was the shortening of both the ejaculation latency and the postejaculatory interval [60]. In agreement with the facilitatory effect of oxytocin on sexual behavior, selective oxytocin receptor antagonists were found to be extremely effective in impairing the copulatory behavior of the above animal species when administered intracerebroventricularly [257,259,260] as well as in abolishing the facilitatory effects on copulatory behavior induced not only by oxytocin but also by other drugs, i.e., apomorphine [261]. Sexual interaction was also found able to increase FOS, the gene product of the immediate early gene *c-fos* in PVN oxytocinergic neurons that projects to spinal cord mediating penile erection ([262] and references therein) (see Section 2 and Section 2.6). Finally, sexual impotence (i.e., the inability of adult male rats to engage in copulation with an ovariectomized estrogen + progesterone-primed receptive female rat) has also been linked in male rats with a low oxytocin mRNA content and increased opioid peptide enkephalin and dynorphin mRNA content in the PVN [119] as well as low levels of NO synthase in the PVN [118], which is in line with a facilitatory effect of oxytocin and NO and an inhibitory effect of opioid peptides at the PVN level in the control of sexual behavior, respectively. In contrast to the above studies cited above, oxytocin has been found to decrease sexual behavior in male prairie voles (see [5]).

Although it is well known that the medial preoptic area not only has a main role in male and female sexual behavior (see [73]), it also contains oxytocin receptors (see [19,20] and references therein), and it was only reported recently that the microinjection of oxytocin into the anterior medial preoptic area improved copulatory activity in sexually experienced male rats, while the microinjection of an oxytocin receptor antagonist impaired a few elements of copulation without any alteration of locomotor activity in the open field. In contrast to expectations, sexually efficient males showed oxytocin receptor binding levels in the anterior medial preoptic area that were lower than those of inefficient animals [19]. One explanation for this inverse relationship between oxytocin receptor binding and sexual efficiency is that the oxytocin receptors may have been internalized or transcriptionally down-regulated in the anterior medial preoptic area of efficient copulators in response to the higher oxytocin levels. These authors have also found that sexual experience is linked to increased oxytocin receptor protein expression in the medial preoptic area [20].

Together, these results led to the suggestion that (i) although it is not required for the manifestation of male sexual behavior, oxytocin in the medial preoptic area, is able to improve copulation and sexual efficiency in sexually experienced male rats [19], and (ii) a mutual interaction exists between central oxytocin and behavior, in which oxytocin improves copulation, and copulation activates the oxytocin/oxytocin receptor system in the medial preoptic area [20].

In spite of the majority of the studies cited above, which support a facilitatory role of oxytocin in male sexual behavior in rats, mice, hamsters, rabbits, and also in dominant squirrel monkeys, oxytocin gene deletion leads to oxytocin knockout mice that mate and copulate normally, as if oxytocin was not required for the expression of these behaviors [264,265], thus further confounding and complicating the putative facilitatory sexual effect of this neuropeptide. However, this is not surprising, as the deletion of other genes that produce neurotransmitters, neuropeptides, and/or neuromodulators known to play a role in sexual behavior also produce knockout mice that mate and copulate normally. Among these are (i) the neuronal NO synthase knockout mice [266], e.g., animals that lack the enzyme that produces NO, one of the primary physiological mediators of penile erection at the local level (cavernous corpora) [246,247] and at the central level, in the PVN [112,117,118], and in the ventral tegmental area [88,89,168] and (ii) the hypogonadal mice, an example of nature’s knockout because these mice bear a specific deletion in the luteinizing hormone-releasing hormone (LHRH) encoding gene, which results in the fact that no LHRH is detectable in the brain of these mice (see [267]). Thus, it is likely that these findings in knockout animals indicate an important characteristic of reproductive physiology, i.e., the redundancy of the systems deputed to its control at central and peripheral level. This redundancy certainly has an evolutionary origin since it assures the passage of genes to the next generation for the survival of the species. Therefore, the fact that the deletion of the oxytocin or NO synthase gene or the mutations in the LHRH gene does not modify the reproductive function and behavior may simply indicate that oxytocin, NO, and LHRH are only three mediators of those working in the numerous systems that control this complex function rather than indicating no role for oxytocin, NO, or LHRH in sexual behavior. Irrespective of the fact that oxytocin gene deletion leads to oxytocin knockout mice that mate and copulate normally, these mice show different anomalies in social behaviors and social interaction, including male aggression and mother–offspring interaction. In addition, they show novel physiological alterations such as obesity and dysfunction in body temperature control when exposed to cold [268,269].

### 3.2. Oxytocin and Female Sexual Behavior in Laboratory Animals

Oxytocin also induces a facilitatory effect on sexual behavior in female rats, mice, and hamsters. Accordingly, in receptive (ovariectomized estrogen + progesterone-primed or even intact) female rats, oxytocin injected intracerebroventricularly increased both the proceptive and receptive (lordosis) elements of female sexual behavior when exposed to sexually potent male rats [61,62,270,271,272]. This sexual facilitatory effect of oxytocin is estrogen- and progesterone-dependent [61,62,273]. Indeed, estrogen and progesterone are well known to increase brain oxytocin content and receptors across the brain [62,274,275,276,277,278]. As found in male rats, the sexual effects of oxytocin on female rats are reduced by oxytocin antagonists injected intracerebroventricularly [148,257,270,279] or into the medial preoptic area [280] and by the injection of an antisense oligonucleotide to the oxytocin receptors infused into the ventral medial nucleus of the hypothalamus [281,282]. In the latter nucleus oxytocin is thought to modulate the sexual behavior of female rats that is controlled by the cyclic variation of ovarian hormones, by participating in the cyclic fluctuations of dendrite remodelling behavior [283]. In fact, during the 4 days of the female rat estrous cycle, oxytocin-labelled dendrites in the ventromedial nucleus show remodelling characterized by a cyclic increase in the number of synaptic contacts [284]. The ventral tegmental area is another brain area where oxytocin facilitates progesterone + estrogen—but not estrogen alone—induced lordosis, and this effect was eliminated by a selective oxytocin receptor antagonist injected into this brain area before oxytocin was [285]. Sexual behavior is also able to increase FOS expression in the oxytocinergic neurons of the medial preoptic area, ventromedial nucleus and PVN, but not the SO of female rats in either normal conditions [286], as found in male rats [262], or in the presence of a male rat that had been sexually conditioned to be a preferential mate [287]. Oxytocin concentration is also increased in the PVN dialysate of female rats primed with estrogen and progesterone during mating. This increase is also higher during sexual activity in under-paced but not unpaced mating conditions [288]. Estrogen + progesterone priming was found to be anxiolytic in the above experiments, as found with mating [289], and this anxiolytic effect decreased due to an oxytocin receptor antagonist injected intracerebroventricularly. These finding led to the suggestion that oxytocin participates in the anxiolytic effect of paced mating [288].

Oxytocin improves also female receptivity in ovariectomized progesterone + estradiol-primed female mice when injected systemically [257] and in ovariectomized estradiol-primed syrian hamsters when injected into the medial preoptic area and/or ventromedial nucleus of the hypothalamus. These effects were abolished by the blockade of the oxytocin receptors present in these brain areas [290,291] but not in sexually experienced prairie voles (see [5,16]), which is in agreement with experiments showing that oxytocin does not improve but rather reduces male sexual behavior in prairie voles (see [16]). However, oxytocin given subcutaneously to sexually naïve female prairie voles is able to reproduce the effects of social contact and to improve sexual behavior in these animals as well [292]. In this regard it is noteworthy that, contrary to polygynous mammals (i.e., rats and mice), female prairie voles do not show spontaneous estrus cycles, and in sexually naive female prairie voles, social interaction with an unfamiliar male triggers an endocrine cascade leading to social bonding and sexual behavior. Accordingly, social contact with and urinary cues from an unfamiliar male stimulate the release of GnRH, which causes the release of LH. LH, in turn, activates the ovaries to produce estrogen and to induce sexual receptivity, which usually occurs within 48 h from the beginning of contact with the male [293,294,295].

As already discussed for the role of oxytocin in the sexual behavior in male laboratory animals, in spite of the majority of the studies cited above, which support a facilitatory role of oxytocin in female sexual behavior in rats, mice, and hamsters (but not in prairie voles), oxytocin gene deletion produces homozygous female oxytocin knockout mice that show normal mating and parturition but with a significant milk letdown impairment, making it seem as if oxytocin is necessary for lactation but not for mating and parturition [264,265].

However, more recent studies show that homozygous female oxytocin knockout mice show a decrease in lordosis together with a rearrangement of dendritic spines in the medial amygdala [296,297], which is in line with a facilitatory role of oxytocin in female sexual behavior. Finally, experiments aimed at examining the olfactory preference for a sexual partner’s odor and direct social interaction in an enriched condition in homozygous female oxytocin knockout mice suggest that oxytocin is necessary for conspecific odor preference and for controlling the initiation of female but not male sexual behavior in mice [298].

## 4. Oxytocin, Sexual Motivation and Sexual Arousal

It is now recognized that oxytocin facilitates both the anticipatory and consummatory phases of sexual behavior. Since oxytocin facilitates penile erection (a main consummatory element of male sexual behavior) in male rats [58,59,87] and the most evident effect of oxytocin on copulatory behavior is a reduction in the post-ejaculatory interval in male rats [60,255], it was first supposed that the neuropeptide mostly facilitates sexual performance. This supposition was also due to the fact that the above studies did delineate between the effects of oxytocin on the anticipatory or consummatory phases of sexual behavior and that, among male rat copulatory parameters measured (mount, intromission and ejaculation latencies, number of intromissions and of ejaculations, postejaculatory interval), only mount latency produced details on modifications in sexual motivation and/or arousal. Indeed, a decrease or an increase in this parameter is thought to indicate an increase or a decrease in sexual motivation, respectively. The other parameters give information on the consummatory phase of sexual behavior or on the velocity at which copulation takes place. However, the role of oxytocin in sexual arousal and motivation became clear when oxytocin receptor antagonists were reported to be very efficacious in abolishing noncontact erections [103], which are believed to be an index of sexual arousal (see [99,117] and references therein). Moreover, it has been recently shown that sexually experienced Long–Evans rats that have been administered the non-peptide oxytocin receptor antagonist L368899 at a dose of 1 mg/kg into the intraperitoneal cavity 40 min before being placed into the center chamber of a three-chambered arena prepared to measure sexual motivation show a decrease in sexual motivation when compared to vehicle-administered controls, with only minor changes in sexual performance (e.g., in copulatory parameters), which is in line with a facilitatory effect of oxytocin and its receptors on sexual motivation [299]. The role of oxytocin in sexual motivation has been definitively proved by the ability of oxytocin to activate mesolimbic and mesocortical dopaminergic neurons that originate in the ventral tegmental area and project to the nucleus accumbens and medial prefrontal cortex. Oxytocin induces this effect directly by stimulating receptors located in the cell bodies of mesolimbic/mesocortical dopaminergic neurons in the ventral tegmental area or indirectly by stimulating the receptors present in the ventral subiculum of the hippocampus or in the posteromedial complex of the amygdala. Accordingly, when given in these two brain areas at a dose that facilitates penile erection, oxytocin causes the activation of glutamic acid neurotransmission in the ventral tegmental area (see [88,89,90,123,167,171,180,181,300]). In line with the above studies, the activation of the D2-type PVN dopamine receptors by dopamine receptor agonists (i.e., apomorphine), causes penile erection and increases extracellular dopamine concentration in the nucleus accumbens, with both responses being eliminated by d(CH_2_)_5_Tyr(Me)^2^-Orn^8^ vasotocin given intracerebroventricularly [171] or directly in the ventral tegmental area [167]. Together, the results of the above studies allowed the proposal that a neural circuit links the PVN with the ventral tegmental area, hippocampus, amygdala, and nucleus accumbens and from one or more of these areas through yet to be identified pathways back to the PVN to control the oxytocinergic neurons that project to the spinal cord (mediating penile erection) or to the ventral tegmental area and/or hippocampus, amygdala, and bed nucleus of the stria terminalis (controlling mesolimbic/mesocortical dopaminergic neurons) (see Figure 7). It is reasonable to propose that this neural circuit, in which oxytocin participates and interacts with many other neurotransmitters and/or neuropeptides (Table 3), plays a role in the integration of the neural activities that control the consummatory (erectile–ejaculatory) and anticipatory (motivational and rewarding) aspects of male sexual behavior in physiological contexts. In line with this hypothesis, extracellular dopamine and its metabolite DOPAC (3,4-hydroxyphenylacetic acid) increase in both the nucleus accumbens and PVN of sexually potent male rats put in the presence of an inaccessible receptive female rat, when noncontact erections take place, and even more so during copulation (e.g., when in copula penile erections take place) [101]. Thus, this neural circuit, while participating in the consummatory aspects of sexual behavior, may also influence mesolimbic/mesocortical dopaminergic neurons and provide a neural substrate at the same time to explain the well-known rewarding features of sexual activity (see [148,301,302]). Accordingly, mesolimbic/mesocortical dopaminergic neurons play a main role in the motivational and rewarding features of natural reinforcing stimuli, i.e., food, water, and sexual activity (see [301,303,304]). In particular, the dopamine released from these neurons is believed to mediate the transposition of motivational aspects of natural stimuli into goal-directed behaviors, which, in the case of sexual activity, may be the seeking of a sexual partner or of sexual intercourse to obtain reward and satisfaction (see [305]). This complex neural circuit that includes mesolimbic, mesocortical and incerto-hypothalamic dopamine, glutamic acid, and central oxytocin neurons among others, which interconnect the above brain areas, may constitute a neural basis to explain the facilitatory involvement of oxytocin in socio-sexual interactions. This involvement was first described in intact laboratory animals, mainly rodents (see [6,306]), and was confirmed later in oxytocin- and oxytocin receptor-knockout mice, which present important deficits in social interaction due to the lack of oxytocin and/or its receptors [190,264,265,268,307,308,309]. These results, which have been now extended to humans with intranasal oxytocin, indicate a role of this ancient neuropeptide in numerous features of human social interaction, from empathy (emotional and cognitive) and decision making to emotional face recognition, trust, and also in the alterations in social interaction seen in mental pathologies, such as in schizophrenia, autism, drug abuse, and addiction, as evidenced by the exponentially increasing number of studies that appear on these topics in scientific literature medlines (see [32,33,40,46,47,48,188,189,191] and references therein). Unfortunately, numerous criticisms have been raised on the enormous number of studies that have been produced, as so far, these studies have not been successfully translated for use in human research in healthy people and in patients affected by mental pathologies (see [38,39])**,** making it difficult to trust this research on intranasal oxytocin in humans.

## 5. Oxytocin and Sexual Behavior in Men

In view of the findings obtained in laboratory animals and that was reviewed above, a facilitatory effect of oxytocin on male sexual behavior may also be present in humans since oxytocin is increased in plasma during sexual activity, mainly at ejaculation [55,56], and during the manipulation of breast and genitalia, which usually happens during sexual intercourse [57]. However, findings supporting this hypothesis are scarce, apart from a report on an improving the effect of systemic oxytocin in a few patients affected by psychogenic impotence [54]. This view has not been modified by intranasal oxytocin, a route of administration that should allow oxytocin to cross the blood–brain barrier and reach the central nervous system (see Section 1). In fact, this treatment has also been reported to be ineffective in modifying either the time to ejaculation or seminal parameters in healthy men when administered before masturbation for ejaculation for semen donation [310]. Intranasal oxytocin has also been found to be inefficacious on sexual arousal and orgasm induced by an erotic film and masturbation as well as the parameters of appetitive, consummatory, and refractory sexual behavior determined with the Acute Sexual Experience Scale in a double-blind, placebo-controlled cross-over design [311]. Intranasal oxytocin (24 IU) was also found to be scarcely active on sexual drive, arousal, penile erection, lubrication, orgasm, and refractory aspects of sexual behavior and on biomarkers such as cortisol, α-amylase, and heart rate together with partner interactions when administered to healthy heterosexual couples in a naturalistic setting; these data were analyzed using the Acute Sexual Experience scale and the Arizona Sexual Experience scale. Nevertheless, analysis of variance (ANOVA) and a hierarchical linear model revealed a few peculiar effects in terms of size, ranging from small to moderate variance linked to the orgasmic/post-orgasmic interval as well as the parameters of partner interactions. As suggested by the hierarchical linear model analysis, oxytocin increased the intensity of orgasm and contentment after sexual intercourse, with these effects being shown to be more marked in men compared to women by ANOVA analysis. With oxytocin administration, men reported also higher levels of sexual satiety after sexual intercourse. Biomarker analysis (cortisol, α-amylase, and heart rate) also indicated moderate psychophysiological activation, but these were not significantly affected by intranasal oxytocin [312]. However, against the conclusions of the above studies, which show very modest effects of intranasal oxytocin on sexual intercourse, a dramatic enhancement in several elements of sexual behavior (libido, erection and orgasm) produced by intranasal oxytocin in a patient under individual treatment for several problems, including social avoidance and relational problems, has been recently reported [313]. In this patient, biopsychosocial evaluation allowed medical conditions and substance-related complications to be excluded as sources of sexual problems [313]. Thus, whether oxytocin may actually improve sexual behavior in men is still far from being assessed.

## 6. Oxytocin and Sexual Behavior in Women

As already discussed for sexual behavior in men, a facilitatory effect of oxytocin on sexual behavior may also be present in women since oxytocin has been found to be increased during sexual activity, mainly by the manipulation of breast and genitalia, which happens during sexual intercourse [57]. However, findings supporting this hypothesis are very limited even today when intranasal oxytocin has become available since this treatment has been also found to be inefficacious in changing sexual arousal and orgasm and the parameters of appetitive, consummatory, and refractory sexual behavior determined together with partner interactions with the Acute Sexual Experience scale in a double-blind, placebo-controlled cross-over design [312]. Through ANOVA analysis, the main finding of this study revealed that women reported to feel more relaxed, and some also referred to better sexual desire sharing capacities or the capacity to be more empathic to their partners. Lubrication was not modified, and biomarkers such as cortisol, α-amylase, and heart rate were only slightly activated but not influenced by oxytocin when compared to the placebo group [312]. The idea that intranasal oxytocin does not influence sexual behavior or the parameters of sexual activation in healthy women has been confirmed by another study showing that intranasal oxytocin (24 IU) does not influence the subjective and objective parameters of sexual drive, arousal, orgasm, and refractory components of sexual behavior or the physiological parameters of sexual activation when measured with vaginal photoplethysmography as well in a double-blind, placebo-controlled, crossover laboratory setting [314]. Similarly to the above studies conducted in healthy women, long-term intranasal oxytocin (32 IU) and placebo in a randomized, prospective, double-blind, placebo-controlled, crossover that lasted 22 weeks were both found to be able to ameliorate sexual activity and depression symptoms in women over time with no treatment, sequence (placebo first/second), or interaction effect [315]. Although studies in a more naturalistic setting are necessary to confirm that oxytocin does not facilitate sexual behavior in women, the data available so far do not support a facilitatory role of the peptide in female sexual behavior.

## 7. Oxytocin, Erectile Function and Sexual Behavior: A Synopsis

Studies on the role of oxytocin in erectile function and sexual behavior show that this neuropeptide controls erectile function and facilitates copulatory behavior in rats and mice by acting mainly in the PVN. Here, oxytocin activates its own neurons by acting on uterine-type oxytocin receptors that, when activated, increase Ca^2+^ influx into the cell bodies of its own neurons. This causes the activation of NO-synthase located in the cell bodies of PVN oxytocinergic neurons [104,316], including those that project to extra-hypothalamic brain areas and the spinal cord [105,317], thus increasing NO production. Newly formed NO activates oxytocinergic neurons to release oxytocin in the extra-hypothalamic brain areas, medulla oblongata, and spinal cord to induce penile erection (see [8,9,64,68,85]) with a mechanism that is currently unknown but that is unrelated to guanylate cyclase and cyclic GMP (see [318]) (Figure 2, Table 2).

Mechanisms similar to those reported above take place in the PVN in physiological contexts, that is, during noncontact erections, copulation, or when oxytocinergic neurons are activated by other neurotransmitters or neuropeptides present in the PVN that facilitate erectile function and/or copulatory behavior (i.e., dopamine, glutamic acid, ACTH-MSH peptides, etc.) (Table 2). Accordingly, (i) NO production increases in the PVN of sexually potent male rats during noncontact erection in the presence of an inaccessible receptive female or during copulatory behavior [117], and (ii) impotent male rats have NO-synthase mRNA levels in the PVN and only have half of those of sexually potent male rats [118]; this decrease is parallel to a decrease in PVN oxytocin mRNA levels and to an increase in opioid mRNA peptide levels, respectively [119].

The activation of oxytocinergic neurons in the PVN causes not only the release of oxytocin in the spinal cord controlling penile erection but also in the ventral tegmental area, the hippocampus, the amygdala, and possibly in the bed nucleus of the stria terminalis, which are all areas that contain the oxytocinergic receptors [161,162,163,182] in which the injection of oxytocin induces penile erection [23,24,89,167,168,180]. These oxytocinergic neurons interconnect the hypothalamus, which has a key role in erectile function and the consummatory components of sexual behavior, and with the limbic system, which plays a main role in the anticipatory phase of sexual behavior (Figure 7). Accordingly, oxytocin in the ventral tegmental area increases the activity of the mesolimbic/mesocortical dopaminergic neurons that project to the nucleus accumbens and medial prefrontal cortex. Dopamine released in these areas induces the activation of incerto-hypothalamic dopaminergic neurons, which impinge on the cell bodies of the PVN-oxytocinergic neurons that control penile erection by mechanisms that have not been identified yet [88,90,171,181]. As found in the PVN, the activation of mesolimbic-mesocortical dopaminergic neurons by oxytocin in the ventral tegmental area is mediated by an increased Ca^2+^ influx into the cell bodies of dopaminergic neurons, causing the activation of NO synthase (see above). However, in contrast to the PVN, in the ventral tegmental area, newly formed, in turn, NO activates a guanylate cyclase-increasing cGMP concentration that leads to the activation of mesolimbic dopaminergic neurons. Accordingly, (i) the inhibition of guanylate cyclase in the ventral tegmental area eliminates ventral tegmental area oxytocin-induced penile erection [168], and (ii) 8-Br-cGMP, an active phosphodiesterase resistant cGMP analogue, induces penile erection when injected into the ventral tegmental area. In line with a facilitatory role of the NO-cGMP signalling system in controlling penile erection in the ventral tegmental area, phosphodiesterase inhibitors clinically used for the therapy of erectile dysfunction (i.e., sildenafil and vardenafil), increase both noncontact erections and extra-cellular dopamine in the nucleus accumbens dialysate when injected into the ventral tegmental area [170].

The mesolimbic/mesocortical dopaminergic neurons in the ventral tegmental area are also activated by oxytocin released in the ventral subiculum of the hippocampus and in the posteromedial cortical nucleus of the amygdala. Accordingly, (i) oxytocin injected into these brain areas activates glutamatergic neurotransmission in the ventral tegmental area, leading to the increased activity of mesolimbic/mesocortical dopaminergic neurons and penile erection, and (ii) penile erection induced by oxytocin injected into the ventral subiculum or posteromedial cortical nucleus of the amygdala occurs concomitantly, resulting in an increase in extracellular dopamine in the nucleus accumbens [89,167]. The available results suggest that oxytocin acts in the ventral subiculum and the posteromedial cortical nucleus by increasing NO production in these areas. NO, in turn, increases glutamic acid neurotransmission in the ventral subiculum, causing penile erection [180,181], possibly with the activation of neural (glutamatergic) efferents projecting from the ventral subiculum directly to the ventral tegmental area or indirectly to extra-hippocampal brain areas (i.e., prefrontal cortex or the nucleus accumbens itself) (see [201,319,320]), modulating the activity of mesolimbic dopaminergic neurons (Figure 7). It is also possible that the posteromedial cortical nucleus works in concert with the ventral subiculum. Accordingly, these two areas are known to modulate the activity of mesolimbic dopaminergic neurons with direct and indirect (i.e., through the medial prefrontal cortex) glutamatergic efferents that reach the nucleus accumbens and/or ventral tegmental area [198,201,319,320,321,322,323,324,325].

The bed nucleus of the stria terminalis BNST is the last brain area that has been discovered so far that receives oxytocinergic projections from the PVN and in which the injection of oxytocin induces penile erection [23]., Here, oxytocin induces penile erection by increasing the release of glutamic acid and dopamine. Glutamic acid, and perhaps dopamine also, activate the NO synthase-containing glutamatergic neurons projecting back to the PVN in turn, leading to the activation of spinal oxytocinergic neurons mediating penile erection in these brain areas, as described above (Figure 7 and [23,24]).

Finally, oxytocin also facilitates penile erection by acting in the lumbo-sacral spinal cord. Accordingly, oxytocin given intrathecally in anaestethized male rats in the lumbo-sacral, but not thoraco-lumbar spinal cord, raises intracavernous pressure, an effect that is eliminated by the oxytocin receptor antagonist d(CH_2_)_5_-Tyr(Me)^2^-Orn^8^-vasotocin and by a section of the pelvic nerves as well [80,202]. In terms of how oxytocin acts in the lumbo-sacral spinal cord to induce penile erection, it is known only that oxytocinergic neurons form synaptic contacts in the dorsal horn preganglionic sympathetic and parasympathetic cell columns in the thoraco-lumbar and lumbo-sacral tract with the spinal neurons innervating penile cavernous corpora [8,9,78,80,202,203]. Here, oxytocin is believed to act as a potent activator of the spinal pro-erectile neurons that project to the cavernous corpora [80,202]. Accordingly, the electrical stimulation of the dorsal nerve of the penis in anaesthetized male rats activates oxytocinergic neurons in the PVN [204,205,206], and the stimulation of the PVN, but also of the medial preoptic area, induces a raise in intracavernous pressure [207,208,209,210]. At the level of the lumbo-sacral spinal cord, oxytocin might activate the spinal pro-erectile neurons not only directly but also by facilitating the proerectile effect of serotonin mediated by 5HT_2C_ receptors (and of drugs that activate 5-HT_2C_ receptors) [214]. Alternatively, oxytocin might also activate spinal descending serotoninergic neurons by acting in the nucleus paragigantocellularis of the reticular formation in the medulla oblongata, where these neurons originate [8,211,216] (see also [214]). Oxytocin in the lumbosacral spinal cord is also important for ejaculation [221]. In fact, oxytocin receptors, which are activated by oxytocin released by spinal PVN oxytocinergic neurons, are expressed in the gastrin-releasing peptide containing neurons of the spinal cord ejaculation generator, whose release controls ejaculation by acting in lumbar and sacral autonomic and motor nuclei, which innervate bulbocavernous and ischiocavernous striated muscles at the base of the penis [222,223,224,225,226,227].

Whether the above mechanisms involving oxytocin identified in male rats also occur in female rats is unknown. However, in numerous studies, oxytocin also facilitates sexual behavior in receptive (ovariectomized estrogen + progesterone-primed or even intact) female rats when given centrally by increasing the proceptive and receptive (lordosis) elements of female sexual behavior with sexually active male rats [60,61,62,270,271,272]. The medial preoptic area and ventromedial nucleus of the hypothalamus are the main sites where oxytocin acts to facilitate female sexual behavior [280]. Accordingly, the effects of oxytocin on the sexual behavior of female rats were prevented by oxytocin receptor antagonists given intracerebroventricularly [148,257,270,279] or into the medial preoptic area [280] and by an antisense oligonucleotide to the oxytocin receptors infused into the ventral medial nucleus [281,282]. Here, oxytocin is thought to modulate female rat sexual behavior controlled by the cyclic variation of ovarian hormones, by participating in the cyclic fluctuations of dendrite remodelling behavior [283]. However, the exact mechanism activated by oxytocin in the medial preoptic area and in the ventromedial nucleus of the hypothalamus to facilitate the proceptive and lordotic components of female sexual behavior is far from being understood. Nonetheless, experimental findings show that neurons that originate in the ventromedial nucleus and project to the periaqueductal gray and from here to the lumbar spinal cord, which controls he lordosis-producing muscles, are activated after oxytocin injection (see [326]).

The data reviewed above show that oxytocin neurons originating in the PVN and surrounding periventricular region, which send their projections across the brain and also to the spinal cord, participate with other neural pathways in a complex neural circuit that reciprocally interconnects the hypothalamus and several brain areas of the limbic system, the medulla, and the spinal cord. This circuit plays a role in the integration of the neural activities controlling the consummatory (erectile–ejaculatory) and anticipatory (motivational and rewarding) components of male sexual behavior in physiological contexts. Although experimental data in female rats are scarce, it is likely that this circuit also plays a role in proceptive and receptive female sexual behavior, as a similar distribution of central oxytocinergic neurons is present in male and female animals. This circuit not only contributes to the consummatory aspects of sexual behavior (erectile function and copulation in males and lordosis in females) but at the same time also increases the activity of the mesolimbic/mesocortical dopaminergic neurons supplying a neural substrate to explain the motivational and rewarding properties of sexual activity (Figure 7, see [148,301,302]). Indeed, dopamine released from these neurons is believed to mediate the transposition of the motivational aspects of natural stimuli into goal directed behaviors, for instance in the case of sexual activity, seeking a sexual partner, and of sexual intercourse, to obtain reward and satisfaction [305].

Perhaps, more importantly, plasma oxytocin is found to be increased by the manipulation of breast and genitalia, which happens during sexual intercourse [57] and during sexual activity in men and women, mainly at ejaculation and orgasm [55,56]. This makes it feasible that this complex neural circuit identified in rats, which plays a role in the integration of the neural activities that control the consummatory and anticipatory components of male sexual behavior in physiological contexts, may also be present and functional in men and women. However, the translation of the neurochemical and behavioral results obtained in rodents reviewed above has not been easy to see in humans, at least so far. In fact, data supporting this possibility are scarce in both men and women. This is true even now, when the recent use of intranasal oxytocin, a route of administration that should allow oxytocin to cross the blood–brain barrier and reach the central nervous system (but see Section 1), has become available and has been extensively used in human studies. Indeed, intranasal oxytocin has been reported to be ineffective in modifying either the time to ejaculation and seminal parameters in healthy men when administered before masturbation for ejaculation for semen donation [310] and sexual arousal and orgasm induced by an erotic film and masturbation as well as in modifying the parameters of appetitive, consummatory, and refractory sexual activity in a double-blind, placebo-controlled cross-over design [311]. Intranasal oxytocin has also been found to be ineffective in modifying sexual arousal, orgasm, and the parameters of appetitive, consummatory, and refractory sexual behavior determined together with partner interactions in a double-blind, placebo-controlled cross-over design in healthy women [312] and was unable to improve sexual activity and depression symptoms when chronically administered in women over time [315,327].

## 8. Final Remarks

In conclusion, although studies in more naturalistic settings are necessary to confirm that oxytocin does not facilitate sexual behavior in men and women, the data available so far do not support a facilitatory role of oxytocin in human sexual behavior. As admitted by other researchers, the involvement of oxytocin in several components of human social interaction, from empathy (emotional and cognitive) to trust and even in the alteration of these aspects found in mental pathologies (from schizophrenia, autism, and depression to drug abuse and addiction) remains elusive and difficult to reproduce [27,28,38,39]. The lack of reproducible study results is a very important problem, as this makes it impossible to confirm that a given effect that has been reported is truly due to oxytocin treatment. This also seems true for research on the role of oxytocin in human sexual behavior since translating the impressive amount of data on oxytocin and sexual behavior accumulated in laboratory animals to humans has proven to be very difficult. Hence, confirming that oxytocin will have some utility in human (male and female) sexual dysfunctions therapies appears to be difficult at the moment, at least on the basis of the available results reviewed here, which only a very modest if any effect at all of intranasal oxytocin treatment on sexual behavior and its parameters in men and women. This is certainly true if we assume that oxytocin given intranasally reaches its receptors in the brain intact. This point is difficult to prove, and it is well known that oxytocin may be rapidly metabolized by the peptidases present in blood [328] as well as in brain tissues (see [329,330,331]) to oxytocin fragments that are devoid of typical oxytocin bioactivity or that induce effects that are different from those induced by intact oxytocin (see [63,330,331] and references therein). This raises the possibility that many of the effects reported after intranasal oxytocin administration are due to some of its fragments not acting on or that are acting on sites that are different from the oxytocin receptor sites. One way to overcome this problem may be to replace oxytocin with synthetic non-peptide oxytocin agonists devoid of the toxic effects that penetrate the blood–brain barrier, accumulate in brain tissues, and act as selective agonists of the oxytocin receptor. Some non-peptide oxytocin agonists are available (see [332]), and a few of them have also been tested in preclinical studies in laboratory animals in the attempt to confirm some of the numerous effects attributed to the neuropeptide [333,334,335]. Unfortunately, to our knowledge, none of these compounds have been tested for their effect on erectile function and sexual behavior yet, although this strategy has already been used to block central oxytocinergic receptors with orally active non-peptide oxytocin receptor antagonists that penetrate the blood–brain barrier and accumulate in the brain after systemic administration such as L-368899 to determine the distribution of oxytocin receptors across the brain [179] and epelsiban (GLK 557296) [229] in the attempt to treat premature ejaculation [230,231]. Hence, it seems reasonable to assume that when safe, orally active and brain accumulating synthetic non-peptide oxytocin agonists devoid of collateral and/or toxic effects will be identified and become available, and it will be possible to ascertain whether oxytocin facilitates sexual behavior in men and women.

In addition to the use of safe orally active and brain accumulating synthetic non-peptide oxytocin agonists, other strategies may be also considered in order to verify whether oxytocin exerts a facilitatory effect on sexual behavior in humans when given systemically and intranasally in particular. Among these strategies are certainly those aimed at increasing the passage of intact oxytocin from the nose to the brain across the nasal epithelium. This might be obtained with the use of newly developed drug-delivery approaches, such as delivery from microspheres, biodegradable wafers, and colloidal drug-carrier systems (e.g., liposomes, nanoparticles, nanogels, dendrimers, micelles, nanoemulsions, polymersomes, exosomes, and quantum dots) (for a review on this matter, see [336] and enclosed references). In line with this possibility, the intranasal administration of nanoparticle-encapsulated oxytocin has been reported to be more effective and to induce a more long-lasting effect than intranasal oxytocin alone in social interaction tests in mice [337]. Unfortunately, as discussed above for synthetic non-peptide oxytocin agonists, it is unknown whether oxytocin administered with this formulation facilitates sexual behavior or not. Thus, more work is necessary to ascertain if synthetic non-peptide oxytocin agonists or oxytocin delivered intranasally with some of the newly developed drug-delivery approaches recalled above or yet to be identified/realized facilitate sexual behavior in laboratory animals and in humans or not.

That the difficulty of translating the facilitatory sexual effect of oxytocin found in rats and mice to men and women is mainly due to its ability to cross the blood–brain barrier, which is certainly a point that needs to be overcome with the methodologies recalled above (i.e., substitution of the peptide with synthetic non-peptide oxytocin agonists or the use of new approaches for oxytocin delivery). However, other reasons must also be considered. One of these is that the majority of studies that have revealed this facilitatory sexual effect of the neuropeptide were conducted in rats and mice. As mentioned above, rats and mice are used to study sexual behavior because of their availability, well-characterized sequence of copulatory activity, and the ease of quantifying the parameters of this copulatory sequence in males and females. The latter point is the one that allows researchers to prove if sexual behavior is improved or impaired in a specific physiological condition or after the consumption of drugs, which, in sexual behavior experiments, is oxytocin. This is not applicable to men and women, as they do not show the same quantifiable sexual parameters as those measured in rodents in laboratory settings. Nevertheless, it is well known that rats are a predictive model for human sexual behavior, as the majority of the drugs that facilitate erectile function and sexual behavior in rats (including the last discovered phosphodiesterase type V) do so also in humans (see [75] and references therein). Intriguingly, the facilitatory sexual effects of oxytocin found in rats and mice are not seen in male prairie voles, as oxytocin inhibits rather than facilitates sexual behavior in this species [5,16]. Prairie voles are a model for studying monogamy and its neurobiological mechanisms, as it leads to partner preference and pair bonding (sexual attachment), aggression towards strangers, and bi-parental care (see [5,16,17,70]). Since oxytocin is thought to exert a facilitatory role on these monogamy components, including sexual attachment in prairie voles [5,6,21,28,32,70,306,338,339] and in humans as well [21,70,339,340], it would be argued that the mechanisms of sexual attachment found in prairie vole better mirror those present in and that are more relevant to humans than those involved in sexual behavior, which is in view of the contradictory findings on rat and human sexual behavior discussed above. Further studies are required to verify this possibility.

Another reason for translating the facilitatory sexual effect of oxytocin found in rats and mice to humans may be its anxiolytic effect (see [340] and references therein) and/or its ability to facilitate social attachment, as discussed above. This raises the possibility that oxytocin reduces anxiety and facilitate social interaction, decreasing the motivation of rats and mice to engage themselves in sexual behavior with a receptive female. However, this is unlikely, at least in rats and mice, as they usually undergo a few preliminary copulatory tests with a receptive female in order to be habituated to the experimental conditions and to eliminate the effect of novelty, including anxiety, before they are used in the experiments from which definitive sexual data are obtained. It also seems unlikely that the anxiolytic effect of oxytocin is responsible for the difficulty of translating the facilitatory sexual effect of oxytocin found in rats to humans, as in the studies with intranasal oxytocin on sexual behavior in humans reviewed above, the effects were only minor and usually facilitatory when they were found, but no detrimental effects on sexual behavior have been reported [312,314,315].

When the authors of this review isolated and purified the hypothalamic extract of a peptide from a rat that was able to induce penile erection when injected in picomole amounts into the lateral ventricles of male rats in 1985, there was much excitement because of the belief that a new brain peptide had been discovered that would have been useful for the treatment of erectile dysfunction. Surprisingly the amino acid sequence of the “new peptide” turned out to be identical to that of oxytocin [59], one of the first peptides to be sequenced and synthesised in the laboratory (see Section 1). This feeling of excitement was replaced with disappointment, but this was soon overcome by the fact that this discovery led the authors to determine the role of this peptide in the control of erectile function and sexual behavior from then until now, the time for them to retired draws more and more near. Although translating the impressive amount of data on oxytocin and sexual behavior accumulated in laboratory animals to humans appears difficult to translate, it is likely that oxytocin may not be useful for this purpose, and continuing to work on determining the relationship between oxytocin and sexual behavior will contribute to extending our knowledge on the central mechanisms controlling this fundamental behavior. These studies will help to provide the scientific basis for new strategies for the treatment of human sexual dysfunctions.

## Figures and Tables

**Table 1:** Amino acid sequence of oxytocin, vasopressin, and some of their peptidic analogues and the chemical formulas of some non-peptidic agonists and antagonists cited in this review.

Peptide, Non-Peptide	Amino Acid Sequence/Chemical Formula	Action on Oxytocin Receptors	Effect on Erectile Function/Sexual Activity
oxytocin	H-Cys-Tyr-Ile-Gln-Asn-Cys-Pro-Leu-GlyNH_2_	agonist	facilitatory
Arg^8^-vasopressin	H-Cys-Tyr-*Phe*-Gln--Asn-Cys-Pro-*Arg*-GlyNH_2_	agonist at 100 times higher doses	none
d(CH_2_)_5_Tyr(Me)^2^-Orn^8^- vasotocin	*Mpr-Tyr(Me)*-Ile-Gln--Asn-Cys-Pro-*Orn*-GlyNH_2_	antagonist	inhibitory
Pen^1^-Phe(Me)^2^-Tyr^4^- Orn^8^-oxytocin	*Pen-Phe(Me)*-Ile-*Tyr*--Asn-Cys-Pro-*Orn*-GlyNH_2_	antagonist	inhibitory
d(CH_2_)_5_Tyr(Me)^2^-Arg^8^- vasopressin	*Mpr-Tyr(Me)*-Ile-Gln--Asn-Cys-Pro-*Arg*-GlyNH_2_	antagonist at high doses	inhibitory at high doses
L-383,899	1-((7,7-dimethyl-2(S)-(2(S)-amino-4-(methylsulfonyl) butyramido)bicyclo[2.2.1]-heptan-1(S)-yl)methyl) sulfonyl)-4-(2-methylphenyl)piperazine	antagonist	NA
epelsiban (GLK 557296)	(3*R*,6*R*)-3-(2,3-dihydro-1*H*-inden-2-yl)-1-[(1*R*)-1-(2,6-dimethyl-3- pyridinyl)-2-(4-morpholinyl)-2-oxoethyl]-6-[(1*S*)-1-methylpropyl]-2,5-piperazinedione	antagonist	NA *
cligosiban	5-{3-[3-(2-chloro-4-fluorophenoxy)azetidin-1-yl]-5-(methoxy methyl)-4H-1,2,4-triazol-4-yl}-2-methoxypyridine	antagonist	NA *

Different amino acid residues are written in italics. The two cysteins in position 1 and 6 are linked by a disulfide bridge shown with a continuous line. L-368,899, epelsiban (GSK 557296), and glicosiban are nonpeptide oxytocin receptor antagonists. Appropriate references are quoted in the review sections. Mpr = 1(β-mercapto-β,β-cyclopentamethylene)-propionic acid; Pen = Penicillamine. * = Found ineffective on premature ejaculation in men. See Section 2 and Section 2.6 for details and references.

**Table 2:** PVN Oxytocinergic neurons are activated and/or inhibited by numerous neurotransmitters and or neuropeptides present in the rat PVN.

Neurotransmitter, Neuropeptide	Receptor	Mechanism of Action
oxytocin	Uterine type	intracellular Ca^2+^ increase and NOS activation, no GC-cGMP pathway involvement
dopamine	D2 and D4 subtype	intracellular Ca^2+^ increase and NOS activation, no GC-cGMP pathway involvement
glutamic acid	NMDA, some role of AMPA receptors cannot be ruled out	intracellular Ca^2+^ increase and NOS activation, no GC-cGMP pathway involvement
GABA	GABA-A	reduction in intracellular Ca^2+^ increase and NOS activation
opioid peptides	µ subtype	reduction in intracellular Ca^2+^ increase and NOS activation
endocannabinoids	CB1	reduction in GLU transmission, intracellular Ca^2+^ increase and NOS activation
hexarelin peptides	apparently different from the GHS receptor	intracellular Ca^2+^ increase and NOS activation, no GC-cGMP pathway involvement
C-terminal VGF peptides	unknown	intracellular Ca^2+^ increase and NOS activation, no GC-cGMP pathway involvement

Appropriate references are quoted in the manuscript. Abbreviations: NMDA =N-methyl-D-Aspartate; AMPA= α-amino-3-hydroxy-5-methyl-4-isoxazolepropionic acid; GLU = glutamate; GC-cGMP = guanylate cyclase–cyclic guanosine monophosphate; GHS =growth hormone secretagogue; NOS= nitric oxide synthase.

**Table 3:** Oxytocin interacts with several neurotransmitters and/or neuropeptides that influence erectile function and sexual behavior at the level of different brain areas.

Neurotransmitter, Neuropeptide	Effect on Sexual Function	Brain Areas Involved	Main Interaction
oxytocin	facilitatory	PVN, MPOA, VMN, BNST, VTA, VS, amygdala, nucleus para-giganto cellularis, SpC	activation of PVN oxytocinergic neurons; activation of mesolimbic dopaminergic neurons in the VTA; activation of glutamatergic neurons in the VS, amygdala, BNST; activation of pro-erectile neurons in the thoraco-lumbar SpC; activation of serotoninergic neurons projecting to the SpC; activation of MPOA and VMN leading to lordosis in females
dopamine	facilitatory	PVN, BNST, VTA, SN, NAs	activation of PVN oxytocinergic neurons; activation of neurons in the NAs projecting to the PVN; modulation of motor activity at SN level (see [263]).
serotonin	Inhibitory, Facilitatory *	SpC	activation/inhibiton* of oxytocinergic neurotransmission in the SpC
glutamic acid	facilitatory	PVN, BNST, VTA, VS, MPFC amygdala	activation of PVN oxytocinergic neurons; activation of mesolimbic dopaminergic neurons in the VTA and of glutamatergic neurons in the BNST, VS and amygdala
GABA	Inhibitory	PVN, SpC	inhibition of PVN oxytocinergic neurons
nitric oxide	facilitatory	PVN, BNST, VTA, VS, amygdala	activation of PVN oxytocinergic neurons; activation of mesolimbic dopaminergic neurons in the VTA; activation of glutamatergic neurons in the VS, amygdala and BNST
ACTH-MSH peptides	facilitatory	APVH	NAs (but see [66,67])
opioid peptides	inhibitory	PVN	inhibition of PVN oxytocinergic neurons by stimulation of opioid receptors of the µ subtype
hexarelin related peptides	facilitatory	PVN	activation of PVN oxytocinergic neurons, possibly by receptors different from those mediating growth hormone release
C-terminal VGF peptides	facilitatory	PVN	activation of PVN oxytocinergic neurons by receptors yet to be identified.
endocannabinoids	inhibitory	PVN	inhibition of PVN oxytocinergic neurons secondary to the inhibition of glutamic acid neurotransmission by activation of cannabinoid receptors of the CB1 subtype.

Details on the mechanism of action of the above compounds can be found in the review and in the cited References. The structure of hexarelin-related peptides and C-terminal VGF-derived peptides can be found in the dedicated References. Abbreviations: APVH = periventricular hypothalamic area; BNST = bed nucleus of the stria terminalis; MPFC = medial prefrontal cortex; MPOA = medial preoptic area; NAs = nucleus accumbens; PVN = paraventricular nucleus of the hypothalamus; SN = substantia nigra; SpC = spinal cord; VS = ventral subiculum of the hippocampus; VMN = ventromedial nucleus; VTA = ventral tegmental area. * Depending on the receptor subtype involved. NA = not available.
